# Cost-Effectiveness of Pharmacogenomics-Guided Prescribing to Prevent Gene-Drug-Related Deaths: A Decision-Analytic Model

**DOI:** 10.3389/fphar.2022.918493

**Published:** 2022-06-28

**Authors:** Cathelijne H. van der Wouden, Heiralde Marck, Henk-Jan Guchelaar, Jesse J. Swen, Wilbert B. van den Hout

**Affiliations:** ^1^ Department of Clinical Pharmacy & Toxicology, Leiden University Medical Center, Leiden, Netherlands; ^2^ Department of Biomedical Data Sciences, Leiden University Medical Center, Leiden, Netherlands

**Keywords:** pharmacogenomics, cost-effectiveness, drug-related death, adverse drug reactions, precision medicine

## Abstract

**Aim:** Prospective studies support the clinical impact of pharmacogenomics (PGx)-guided prescribing to reduce severe and potentially fatal adverse effects. Drug-gene interactions (DGIs) preventing potential drug-related deaths have been categorized as “essential” by the Dutch Pharmacogenetics Working Group (DPWG). The collective clinical impact and cost-effectiveness of this sub-set is yet undetermined. Therefore, we aim to assess impact and cost-effectiveness of “essential” PGx tests for prevention of gene-drug-related deaths, when adopted nation-wide.

**Methods:** We used a decision-analytic model to quantify the number and cost per gene-drug-related death prevented, from a 1-year Dutch healthcare perspective. The modelled intervention is a single gene PGx-test for *CYP2C19, DPYD, TPMT or UGT1A1* to guide prescribing based on the DPWG recommendations among patients in the Netherlands initiating interacting drugs (clopidogrel, capecitabine, systemic fluorouracil, azathioprine, mercaptopurine, tioguanine or irinotecan).

**Results:** For 148,128 patients initiating one of seven drugs in a given year, costs for PGx-testing, interpretation, and drugs would increase by €21.4 million. Of these drug initiators, 35,762 (24.1%) would require an alternative dose or drug. PGx-guided prescribing would relatively reduce gene-drug related mortality by 10.6% (range per DGI: 8.1–14.5%) and prevent 419 (0.3% of initiators) deaths a year. Cost-effectiveness is estimated at €51,000 per prevented gene-drug-related death (range per DGI: €-752,000–€633,000).

**Conclusion:** Adoption of PGx-guided prescribing for “essential” DGIs potentially saves the lives of 0.3% of drug initiators, at reasonable costs.

## 1 Introduction

Pharmacogenomics (PGx)-guided prescribing promises to personalize drug therapy by using an individual’s germline genetic makeup to guide dose and drug selection (Weinshilboum and Wang, 2004; Relling and Evans, 2015). This ameliorates the conventional ‘trial and error’ approach of drug prescribing, thereby reducing risk of lacking efficacy and adverse drug events (ADRs) (Pirmohamed, 2014). ADRs are a significant burden for individual patients and society and are an important cause of emergency department visits and hospital admissions (Leape et al., 1991; Lazarou et al., 1998; Pirmohamed et al., 2004). The resulting economic burden in the United States has been estimated at $30 billion to $136 billion annually (Johnson and Bootman, 1995). Several prospective studies support the clinical impact of individual gene-drug interactions (DGIs) to either optimize dosing (Pirmohamed et al., 2013; Verhoef et al., 2013; Coenen et al., 2015; Wu, 2015; Henricks et al., 2018) or drug selection (Mallal et al., 2008; Claassens et al., 2019a). Additionally, both the Clinical Pharmacogenetics Implementation Consortium (CPIC) (Relling and Klein, 2011; Relling et al., 2020) and the Dutch Pharmacogenetics Working Group (DPWG) (Swen et al., 2008; Swen et al., 2011; Swen et al., 2018) have developed guidelines on incorporating PGx results into drug prescribing. Appropriate sub-groups have previously been identified for PGx testing, including cardiovascular (Chatzopoulou et al., 2022) (supportive-)oncology (Patel, 2021; Patel et al., 2021), geriatric and polypharmacy patients (Brixner et al., 2016). Nevertheless, ambiguity remains regarding whether and which PGx tests should be prioritized for implementation into routine care (Roden et al., 2018). In an effort to overcome this inconclusiveness and to direct clinicians on requesting relevant PGx tests, the DPWG developed the Clinical Implication Score, where DGIs classified as “essential” direct clinicians to request a single-gene PGx test pre-therapeutically to guide dose and drug selection of the interacting drug (Swen et al., 2018). The Clinical Implication Score is based on the severity of clinical consequences associated with the DGI, the level of evidence for the association, the number needed to genotype to prevent an ADR with Common Terminology Criteria of Adverse Events (CTCAE) grade ≥3, and the level of PGx information included in the drug label. “Essential” DGIs comprise of high-risk drugs and corresponding recommendations intend to prevent severe clinical consequences such as gene-drug-related death. Therefore, they may be considered a minimum list of DGIs for which pre-therapeutic PGx-testing should be performed.

While numerous implementation barriers have been overcome, pre-therapeutic PGx-testing for all “essential” DGIs is not yet routine care and significant barriers preventing adoption remain (Swen et al., 2007; Haga and Burke, 2008; Abbasi, 2016). A prominent barrier is the lack of reimbursement of single-gene PGx tests, despite the availability of numerous cost-effectiveness analyses (Wong et al., 2010; Plumpton et al., 2016). Reimbursement of PGx tests for “essential” DGIs may be supported by studies quantifying the impact and cost-effectiveness of wide-spread adoption. Here, impact on the most severe outcome, mortality, may be most impactful.

Although the incidence of DGIs, when adopted nation-wide, has been estimated (Schildcrout et al., 2012; Samwald et al., 2016; Bank et al., 2019) and the cost-effectiveness of numerous DGIs in single-gene scenarios have been determined (Wong et al., 2010; Plumpton et al., 2016), the collective downstream effect of “essential” DGIs on clinical outcomes and cost-effectiveness after wide-spread adoption remains undetermined. Here, we therefore aim to assess the collective impact and cost-effectiveness of PGx for DGIs categorized as “essential” to prevent gene-drug-related deaths when adopted nation-wide in Netherlands using a decision-analytic model. The decision analytic model bases the risk of gene-drug-related death on literature review, the incidence of drug initiation on Dutch prescription data, and the predicted phenotype category frequencies on a Dutch sample.

## 2 Methods

### 2.1 Study Design

We developed a decision-analytic model to assess the number and cost of gene-drug-related deaths prevented with PGx-guided initial dose and drug selection for “essential” DGIs, among patients initiating potentially interacting drugs in the Netherlands when compared to standard of care in 1 year. DGIs were selected based on the following criteria: 1) the clinical implication score is “essential”, meaning that DPWG advises pre-therapeutic genotyping and 2) the DGI has clinical relevance score F (CTCAE Grade 5) and is therefore associated with gene-drug-related death for at least one predicted phenotype category. These selection criteria yielded the interactions between four genes (*CYP2C19, DPYD, TPMT*, and *UGT1A1*) and seven drugs (clopidogrel, capecitabine, systemic fluorouracil, azathioprine, mercaptopurine, tioguanine, and irinotecan). See Table 1 for an overview of selected gene-drug pairs. When the DPWG recommendations suggested either dose reduction or an alternative drug, this model assumed dose reduction as the intervention.

**TABLE 1:** Selected “essential” gene-drug pairs, their potential consequences and DPWG recommendation per phenotype category.

Drug	Gene	Predicted phenotype	Actionable DGI	DPWG Recommendation	Most Severe Preventable Clinical Consequence Potentially Leading to Gene-Drug-Related death^a^
Azathioprine	*TPMT*	TPMT EM	No	-	-
		TPMT IM	Yes	Dose reduction to 50%	Severe myelosuppression
		TPMT PM	Yes	Dose reduction to 10% or alternative drug	Severe myelosuppression
Capecitabine	*DPYD*	DPYD GAS 0	Yes	Alternative drug	Fluoropyrimidine induced toxicity
		GAS 0.5/PHENO	Yes	Dose adjustment based on DPD phenotype	Fluoropyrimidine induced toxicity
		DPYD GAS 1.0	Yes	Dose reduction to 50%	Fluoropyrimidine induced toxicity
		DPYD GAS 1.5	Yes	Dose reduction to 50%	Fluoropyrimidine induced toxicity
		DPYD GAS 2.0	No	-	-
Clopidogrel	*CYP2C19*	CYP2C19 EM	No	-	-
		CYP2C19 IM	Yes	Dose increase to 200% or alternative drug	
		CYP2C19 PM	Yes	Alternative drug (ticagrelor, prasugrel or dipyridamole)	Cardiovascular death
		CYP2C19 UM	No	-	Cardiovascular death
Fluorouracil	*DPYD*	DPYD GAS 0	Yes	Alternative drug	Fluoropyrimidine induced toxicity
		GAS 0.5/PHENO	Yes	Dose adjustment based on DPD phenotype	Fluoropyrimidine induced toxicity
		DPYD GAS 1.0	Yes	Dose reduction to 50%	Fluoropyrimidine induced toxicity
		DPYD GAS 1.5	Yes	Dose reduction to 50%	Fluoropyrimidine induced toxicity
		DPYD GAS 2.0	No	-	-
Irinotecan	*UGT1A1*	UGT1A1^a^1/*1	No	-	-
		UGT1A1^a^1/*28	Yes	-	-
		UGT1A1^a^28/*28	Yes	Dose reduction to 70%	Severe myelosuppression and diarrhea
		UGT1A1 IM	No	-	-
		UGT1A1 PM	No	Dose reduction to 6%	Severe myelosuppression and diarrhea
Mercaptopurine	*TPMT*	TPMT EM	No	-	-
		TPMT IM	Yes	Dose reduction to 50%	Severe myelosuppression
		TPMT PM	Yes	Dose reduction to 10% or alternative drug	Severe myelosuppression
Tioguanine	*TPMT*	TPMT EM	No	-	-
		TPMT IM	Yes	Dose reduction to 50%	Severe myelosuppression
		TPMT PM	Yes	Dose reduction to 10% or alternative drug	Severe pancytopenia

a Clinical relevance score: CTCAE, 5 (death), as reported in the summary of literature underlying the DPWG, recommendations; DGI, drug-gene interaction; EM, extensive metabolizer; IM, intermediate metabolizer; PM, poor metabolizer; UM, ultra-rapid metabolizer; DPWG, dutch pharmacogenetics working group, and GAS = gene activity score.

### 2.2 Decision Analytic Model

The following model was used to calculate the number of gene-drug-related deaths prevented within 1 year:

$$N_{GDRDP}=\overset{7}{\underset{Drug=1}{\sum }}N_{Drug}\times \sum_{Pheno}P_{Pheno}\times \;\left(AR_{Drug,Pheno}^{SoC}-AR_{Drug,Pheno}^{PGx}\right)$$

N_GDRDP_ = gene-drug-related deaths prevented; N_Drug_ = number of drug initiators; P_Pheno_ = predicted phenotype category; AR = absolute risk of gene-drug-related death within 1 year; SoC = for standard of care; PGx = for pharmacogenomics guided initial drug and dose selection.; Drug, Pheno = for a specific drug and a predicted phenotype category.

The following model was used to calculate the cost of gene-drug-related deaths prevented within 1 year:

$$Cost=\overset{7}{\underset{Drug=1}{\sum }}N_{Drug}\times \sum_{Pheno}P_{Pheno}\;\times \;\left(Cost_{PGx}+Cost_{HCP}+Cost_{Drug,Pheno}^{PGx}-Cost_{Drug,Pheno}^{SoC}\right)$$

N_Drug_ = number of drug initiators; P_Pheno_ = predicted phenotype category; Cost_PGx_ = single-gene test; Cost_HCP_ = physician and pharmacist time for interpretation and discussion of actionable PGx results; PGx = pharmacogenomics guided initial drug and dose selection; SoC = standard of care; Drug, Pheno = for a specific drug and a predicted phenotype category.

Finally, the cost per gene-drug-related death prevented was calculated by dividing cost by the number of deaths prevented both per individual DGI and overall.

### 2.3 Model Inputs

#### 2.3.1 Number of Patients Initiating One of the Seven Drugs in the Netherlands

The number of patients a year initiating each of the seven drugs was estimated by multiplying the yearly number of users by the ratio of initiators and users. The yearly number of users was extracted from the Dutch nation-wide GIP databank from the most recent available year; azathioprine, clopidogrel, systemic fluorouracil and irinotecan from 2018, mercaptopurine and tioguanine from 2017 and capecitabine from 2014 (GIP Databank, 2021). For fluorouracil, only aggregated systemic and cutaneous data are reported in the GIP databank. To exclude the cutaneous users we multiplied total number of users with the percentage of systemic fluorouracil users in the Leiden University Medical Center (LUMC) in 2018. The ratio of initiators and users was extracted per drug from the LUMC electronic medical record (EMR) for 2013 until 2018. Here users were defined as those who had a prescription for that drug in their EMR in this period and initiators were defined as users who lacked a prescription for that drug before 2018. See Supplementary Table S1 for an overview of the used ratios and calculated number of nation-wide drug initiators.

#### 2.3.2 Predicted Phenotype Category Frequencies

The predicted phenotype frequencies for the selected genes were derived from a Dutch sample (*n* = 1,023) (van der Wouden et al., 2019a). The variants tested to determine phenotype have been described in detail (van der Wouden et al., 2019a). The genotypes are translated into predicted phenotype categories based on functionalities as described in the DPWG recommendations (Swen et al., 2008; Swen et al., 2011; Swen et al., 2018).

#### 2.3.3 Risk of Gene-Drug-Related Death

The most severe outcome among patients receiving standard of care, as reported in literature underlying the DPWG recommendations, associated with each “essential” DGI is shown in Table 1. Each DPWG recommendation suggests either a dose adjustment or selection of an alternative drug, to reduce the risk of both gene-drug-related deaths and other less severe ADRs. For our model, we extracted the absolute risk of gene-drug-related death within 1 year both in patients receiving the PGx-informed and standard of care (i.e., PGx uninformed) drug treatments for each predicted phenotype category independently, since the risk of gene-drug-related death varies across predicted phenotype categories. For example, the risk of fluoropyrimidine-induced toxicity increases with decreasing DPYD gene activity scores (GAS). Furthermore, when a PGx test is used to guide dose selection, individuals with an actionable phenotype (DPYD GAS 0–1.5) have a reduced risk of fluoropyrimidine-induced toxicity compared to individuals with an actionable phenotype using a normal dose. On the other hand, the risk of fluoropyrimidine-induced toxicity in individuals with a non-actionable predicted phenotype (DPYD GAS 2) will have the same mortality risk, regardless of being tested, since the dose is the same in both groups. Therefore, we have extracted the absolute risk of gene-drug-related death for each predicted phenotype category from the literature, across three groups: 1) tested-actionables (e.g., DPYD GAS 0, 0.5, 1 and 1.5 with PGx informed reduced dose), 2) non-actionables (e.g., DPYD GAS 2 with normal dose) and 3) untested-actionables (e.g., DPYD GAS 0, 0.5, 1 and 1.5 with normal dose). The actionable drug-gene pairs are categorized in Table 1.

A systematic methodology was used to select relevant publications from publications underlying the DPWG guideline which were suitable for risk extraction and is described in detail in Supplementary Table S2. In brief, six steps are performed chronologically until relevant publications have been selected from which absolute risk of gene-drug-related death for each of the tested and untested predicted phenotype categories can be extracted. The scientific rigor of publications decreases with each step and corresponds to the DPWG quality of evidence score (Swen et al., 2008; Swen et al., 2011). The first two steps select publications powered on mortality, the second two steps select publications powered on intermediate outcomes that are associated with mortality and the last two steps resort to additional literature search or estimation. Risk extraction is performed by using methodology corresponding to that step. Each extracted absolute risk of gene-drug-related death is given a certainty score based on the step in which publications are selected. The certainty score ranges from 4 (very certain) to 0 (very uncertain). An overall certainty score per DGI is calculated by taking the mean of the certainty scores of all tested and untested predicted phenotype categories. The systematic selection of publications and extracted absolute risks of gene-drug-related related deaths are described in Supplementary Table S3.

#### 2.3.4 Predicted Phenotype Category Frequencies

The predicted phenotype frequencies for the selected genes were derived from a Dutch sample (*n* = 1,023). The variants tested to determine phenotype have been described in detail (van der Wouden et al., 2019b). The genotypes are translated into predicted phenotype categories based on functionalities as described in the DPWG recommendations.

#### 2.3.5 Costs

Costs are estimated from a health care perspective, with a 1-year time-horizon, and are reported in Euros. The costs of different single-gene PGx tests were based on single-gene prices set in the LUMC in 2018 and on prices from the Dutch Healthcare Authority (NZa). This includes sample collection, analysis, and report of the predicted phenotype and dosing recommendation to the requesting pharmacist. The pharmacist time to record and discuss results with the physician and patient was set at 18 min. The physician time to discuss results with the pharmacist was set at 6 min. Time spent was multiplied by the hourly salaries of Clinical Pharmacists and Medical Specialists as standardized in Dutch Academic Hospitals in 2019 (Cao universitair medische centra, 2018-2020, 2020). The cost of drugs for both standard of care and PGx-guided treatments was calculated for a time-horizon of 1 year. The applied dose was based on the most common indication for the relevant drug and calculated using a base case of 75 kg and a body surface area of 1.7 m2. The price of drugs was extracted from the national drug price registry (Medicijnkosten.nl, 2021) by selecting the least expensive suitable dose and formulation. See Supplementary Table S4 for an overview of the costs used in the model.

#### 2.3.6 Model Assumptions

The adoption of PGx test requesting among initiators was assumed at 100%, DPWG recommendation adherence was assumed at 100% and the dose of drugs to be as per protocol for the indications which were investigated in publications from which risk data was extracted. Regarding the target population and allele frequencies, the ethnicity was assumed Caucasian, and patients were assumed to use similar comedications as patients enrolled in studies from which risks were extracted.

#### 2.3.7 Funding and Ethical Approval

This study was funded by the European Community’s Horizon 2020 Program under grant agreement No.668353 (U-PGx). The funder played no role in this study’s design, conduct or report. Ethical approval was not required for this analysis. The data inputs are collected from publicly available sources.

## 3 Results

As shown in Table 2, on a population of 17 million Dutch inhabitants, 148,128 patients initiate one of seven drugs in a given year, of which the clopidogrel initiators form the largest group (79.6%).

**TABLE 2:** Overall Costs of PGx-testing, pharmacist and physician time for interpretation and drug treatment.

Drug	N drug Initiators	Cost of PGx Test/€ per Initiator	Average Cost of HCP Interpretation of Actionable PGx Result/€ per Initiator^a^	Average Cost of Drugs for Standard of Care (SoC) Treatment/€ per Initiator in 1 year	Average Cost of Drugs for PGx-Guided Treatment/€ per Initiator in 1 year	Difference in Average Drug Costs^b^ (SoC-PGx) (% Saved)/€ per Initiator in 1 year	Total Costs for all initiators^c^/€
Azathioprine	6,979	132	1	248	237	11 (4.6%)	854,659
Capecitabine	8,860	132	1	1,204	1,158	46 (3.9%)	775,246
Clopidogrel	117,900	132	5	15	38	−24 (−62%)	18,923,430
Fluorouracil (systemic)	6,765	132	1	82	79	3 (4.0%)	880,112
Irinotecan	2,593	66	2	14,842	14,588	253 (1.7%)	-481,019
Mercaptopurine	2,177	132	1	1,956	1,875	81 (4.3%)	114,172
Tioguanine	2,854	132	1	1,088	1,080	7 (0.7%)	359,471
TOTAL for all initiators/€	148,128	19,381,790	586,167	60,519,056	61,977,169	−1,458,113	21,426,070
Mean per initiator/€	-	131	16^a^	409	418	10	145

PGx, pharmacogenomic.

a Note: only those with an actionable drug-gene interaction will be interpreted by an HCP.

b [cost drugsstandard of care]-[cost drugsPGx-guided]*[Ndrug initiators].

c [costPGxtest]+[costpharmacist and physician time]-[costdrugs].

### 3.1 Impact on Costs

The total costs of single-gene PGx-testing, interpretation, and additional drugs would be €21.4 million (mean €145 per patient), of which the relevant single-gene test comprises 90.7% (€19.4 million in total, mean €131 per patient). Of these drug initiators, 35,762 (24.1%) would have an actionable DGI, requiring an alternative dose or drug. Health care professional (HCP) discussion of these actionable results would cost €586,000 (€16 per actionable patient). The extra drug costs made for initiating PGx-guided drug treatment is €1.5 million (€10 per patient), of which €2.4 million additional costs as a result of alternative drug treatment and €941,000 costs saved as a result of dose lowering. Interestingly, PGx-guided drug treatment costs are cost-saving for most DGIs (range per cost-saving DGI: 0.7–4.6%), except the clopidogrel-CYP2C19 interaction where the drug costs are €2.8 million higher (€24 per patient, +162%) than standard of care. For the irinotecan-UGT1A1 interaction, the costs of drugs saved in the PGx-guided group surmounts the cost of PGx-testing and HCP interpretation combined, making the intervention cost-saving with €481,000 on irinotecan drug costs.

### 3.2 Number of Gene-Drug-Related Deaths Prevented

As shown in Table 3, PGx-guided initial dose and drug selection would relatively reduce total gene-drug-related mortality by 10.6% (range per DGI: 8.1–14.5%) and prevent 419 (0.3% of initiators) deaths per year. The average certainty score was 2.5 (fairly certain) when weighed for deaths prevented or for number of patients, and ranged from of 0 (very uncertain) to 3 (certain) for individual DGIs.

**TABLE 3:** Cost-effectiveness of PGx-guided pharmacotherapy for gene-drug interactions to prevent gene-drug-related deaths.

Drug	N drug Initiators	Predicted phenotype	Phenotype Frequency	N actionable DGI^a^	Absolute Risk Reduction/% ^b^	N gene-Drug-Related Deaths with Standard of Care	N gene-Drug-Related Deaths with PGx-Guided Care	N gene-Drug-Related Deaths prevented^c^ (RRR%)	Number needed to genotype (NNG)^d^	Certainty Score	Cost to Prevent 1 GDR death^e^/€
Azathioprine	6,979	TPMT EM	0.912	0	0.00	15.8	13.5	2.3 (14.5%)	3,057	2 (fairly certain)	374,411
		TPMT IM	0.087	607	0.36						
		TPMT PM	0.001	7	0.97						
Capecitabine	8,860	DPYD GAS 0	0.001	9	0.76	22.4	20.6	1.8 (8.1%)	4,863	1 (uncertain)	425,488
		GAS 0.5/PHENO	0.000	0	0.58						
		DPYD GAS 1.0	0.018	157	0.39						
		DPYD GAS 1.5	0.054	481	0.24						
		DPYD GAS 2.0	0.925	0	0.00						
Clopidogrel	117,900	CYP2C19 EM	0.673	0	0.00	3,887.8	3,477.0	410.8 (10.6%)	287	3 (certain)	46,064
		CYP2C19 IM	0.245	28,893	0.30						
		CYP2C19 PM	0.037	4,407	0.05						
		CYP2C19 UM	0.045	0	0.00						
Fluorouracil (systemic)	6,765	DPYD GAS 0	0.001	7	0.76	17.1	15.7	1.4 (8.1%)	4,863	1 (uncertain)	632,612
		GAS 0.5/PHENO	0.000	0	0.58						
		DPYD GAS 1.0	0.018	120	0.39						
		DPYD GAS 1.5	0.054	367	0.24						
		DPYD GAS 2.0	0.925	0	0.00						
Irinotecan	2,593	UGT1A1 *1/*1	0.430	0	0.00	4.7	4.1	0.6 (13.6%)	4,055	2 (uncertain)	-752,191
		UGT1A1 *1/*28	0.466	0	0.00						
		UGT1A1 *28/*28	0.101	261	0.24						
		UGT1A1 IM	0.002	0	0.00						
		UGT1A1 PM	0.001	3	0.24						
Mercaptopurine	2,177	TPMT EM	0.912	0	0.00	4.9	4.2	0.7 (14.5%)	3,057	2 (fairly certain)	160,309
		TPMT IM	0.087	189	0.36						
		TPMT PM	0.001	2	0.97						
Tioguanine	2,854	TPMT EM	0.912	0	0.00	6.5	5.5	0.9 (14.5%)	3,057	0 (very uncertain)	385,084
		TPMT IM	0.087	248	0.36						
		TPMT PM	0.001	3	0.97						
TOTAL	148,128	-	-	35,762 (24.1%)	0.3	3,959	3,541	419 (10.6%)	-	2.5 (fairly certain)	51,187

DGI, drug-gene interaction; PGx-guided = pharmacogenomics guided; RRR, relative reduced risk; GDR, gene-drug-related death.

a [Nactionable DGI]*[Pphenotype]*[Ndrug initiators].

b [absolute risk untestedphenotype]-[absolute risk testedphenotype].

c [N drug initiators]/[NNG].

d 1/(SUMDGI [absolute risk reductionphenotype]*[P phenotype]).

e [total costs]/[Ndeaths prevented].

### 3.3 Cost-Effectiveness Analysis

Preventing 419 gene-drug-related deaths with an increase of €21.4 million in healthcare costs, cost-effectiveness is estimated at €51,000 per prevented gene-drug-related death (range per DGI: €-752,000–€633,000). For the irinotecan-UGT1A1 interaction, PGx-guided treatment reduces both mortality and costs (resulting in a negative cost-effectiveness ratio).

## 4 Discussion

Nation-wide adoption of PGx-guided initial dose and drug selection of “essential” DGIs can potentially save the lives of 419 (0.3% of drug initiators) a year at a cost of €51,000 per prevented death. The weighted average certainty score for this analysis 2.5 (fairly certain). In high-income countries an intervention is considered cost-effective when one gained quality-adjusted life year (QALY) costs less than a threshold between €20,000–60,000 (Nghiem et al., 2017). Since PGx-guided pharmacotherapy prevents gene-drug related deaths, it will contribute numerous QALYs; the magnitude of which is associated with the number of additional years that is gained by preventing the fatal gene-drug associated ADR. The investigated seven drugs are generally used to treat life-threatening diseases, and as a result, if treatment is effective and safe, patients will have a below-average though still considerable life-expectancy. Therefore, the additional cost of €51,000 per prevented death is well under the cost-effectiveness thresholds and can be considered reasonable and cost-effective.

### 4.1 Comparison to Current Literature

To our knowledge, we are the first to quantify both the collective impact and cost-effectiveness of nation-wide PGx-guided initial drug and dose selection for DGIs categorized as “essential” on mortality outcomes. Regarding collective impact, previous efforts have quantified the incidence of DGIs when adopted nation-wide (Schildcrout et al., 2012; Samwald et al., 2016; Bank et al., 2019). Bank et al. estimated that nation-wide adoption in the Netherlands of all DPWG recommendations would result in 23.6% of new prescriptions for PGx drugs would have an actionable DGI requiring adjustment of pharmacotherapy (Bank et al., 2019). However, the downstream impact on clinical outcomes were undetermined. In terms of cost-effectiveness, previous efforts have assessed individual drug-gene interactions but have not assessed the collective cost-effectiveness of “essential” DGIs. These include investigation of HLA-B*57:01 testing before abacavir initiation (Hughes et al., 2004), HLA-B*58:01 testing before allopurinol initiation (Plumpton et al., 2017), HLA-B*15:02 and HLA-A*31:01 before carbamazepine initiation and CYP2C9 and VKORC1 guided initial dosing of warfarin (Eckman et al., 2009). However, these DGIs were not considered “essential” by the DPWG and were therefore not included in our analysis. Consistent with individual DGIs investigated here, previous studies have shown the cost-effectiveness of UGT1A1 for irinotecan dosing (Gold et al., 2009; Butzke et al., 2016), CYP2C19 for clopidogrel dosing and alternative drug selection (Reese et al., 2012; Kazi et al., 2014), and TPMT guided initial dosing for thiopurines (Sluiter et al., 2019). Although a cost-minimization study for DPYD guided dosing has been performed (Henricks et al., 2019; Toffoli et al., 2019), its cost-effectiveness remains undetermined.

### 4.2 Model Design and Inputs

The outcome selected for this decision-analytic model is gene-drug-related death. This outcome excludes other, less severe, outcomes which may be improved by PGx-guided pharmacotherapy such as reduction in non-fatal ADRs or lack of drug efficacy. Excluding less severe but probably more prevalent gene-drug associated ADRs may therefore have resulted in an underestimation of the impact of PGx on patient outcomes. Taking these non-fatal ADRs into account would further confirm the cost-effectiveness of PGx-guided pharmacotherapy for “essential” DGIs. On the other side of the spectrum, while the PGx intervention decreases the risk of gene-drug associated ADRs, it may also increase risk of other negative effects such as loss of efficacy or increased risk for other ADRs. These are excluded from the current analysis and as a result we may have overestimated the (cost-)effectiveness. Regarding loss of efficacy, we expect equal drug exposures and benefit/risk among IMs and PMs receiving reduced doses and EMs receiving normal doses, as prospectively demonstrated (Henricks et al., 2018). The extent to which efficacy may be compromised is largest in drugs with a steep dose-response curve and where the default population dose is not at maximum effect or saturated receptor occupancy (Peck, 2018). Therefore, we do not expect that excluding loss of efficacy has affected our overall results much since efficacy was included in the intermediate outcome (which was a composite of death, cardiovascular death, nonfatal myocardial infarction, and nonfatal stroke) for the most predominant DGI (clopidogrel-CYP2C19). The potential underestimation from excluding potential other ADRs can be illustrated by ADRs associated with the PGx-guided treatment. For example, although CYP2C19 guided treatment for clopidogrel dosing or alternative selection was non-inferior to treatment with ticagrelor or prasugrel at 12 months with respect to thrombotic events, treatment with ticagrelor or prasugrel resulted in higher incidence of minor bleeding (Claassens et al., 2019b). In this particular example, excluding minor bleeding from the mode has not affected the validity of our results, since minor bleeding do not result in drug-related death.

The time-horizon of the decision-analytic model was set at 1 year, consistent with the follow-up duration of the supporting trials. Ignoring impact beyond 1 year may have led to an underestimation of the benefit of the intervention. On the other hand, the imposed time-horizon overestimates the costs saved by the PGx intervention. In our current analysis we observed an overall cost increase for PGx-guided drug therapy when compared to standard of care which was driven by increased costs of PGx-guided alternatives for clopidogrel (increased cost of €2.8 million per year). Since clopidogrel is used life-long after a Transient Ischemic Attack, the additional drug costs will increase with an increasing time horizon. Additionally, we did not take into account potential dose or drug changes which may have occurred within standard of care, in the absence of a PGx test. If these changes were to be made within this 1 year time-horizon there would be no additional effect relative to the PGx intervention. This may be the case for drugs, such as fluoropyrimidines and thiopurines which may be dosed in standard of care upon other biomarkers, such as hematological counts.

Potential factors limiting the generalizability of the model are the underlying assumptions made. Firstly, to facilitate absolute risk extraction, we assumed each of the drug initiators to have one particular indication (as described in Supplementary Table S3) and to receive a corresponding standardized drug dose. However, some drugs included in the analysis can be applied for numerous indications. Patients with these other indications may have a different baseline risk of gene-drug-related death as a result of variation in general health or clinical monitoring. Additionally, the effectiveness of PGx-guided prescribing may also vary across indications due to different applied doses. For example, we performed risk extraction for thiopurines on publications including Inflammatory Bowel Disease patients. However, a minority of patients initiating thiopurines has other indications such as Acute Lymphatic Leukemia or Rheumatoid Arthritis, which are applied at higher doses and among patients who are monitored more closely for myelosuppression. Therefore risk of drug-induced death may be different than those with Inflammatory Bowel Disease. Secondly, we assumed the ethnicity of the target population be Caucasian and therefore limited publication selection for absolute risk extraction to those performed in predominantly Caucasian samples. Since allele frequencies vary across ethnicities, we would be hesitant to extrapolate the reported results to ethnicities not included in the underlying publications. While for TMPT (McLeod et al., 1999) allele frequencies are fairly constant across ethnicities, the frequency of actionable phenotypes are higher for UGT1A1 in Blacks and Hispanics (Leger et al., 2018), CYP2C19 in Asians (Zhou et al., 2017) and DPYD in Africans (Mattison et al., 2006) and therefore the current analysis underestimates cost-effectiveness in these ethnicities. Thirdly, the current model was constructed for the Netherlands. Since the effectiveness of the PGx intervention may be dependent on the quality of the health-care system we would be hesitant to extrapolate our results to counties with a different quality of health-care system. If both the healthcare system and ethnicity is similar, we would suggest extrapolating our results to other countries in proportion to the population size (17 million).

In this study, we estimated the number of drug initiators of the investigated seven drugs to be 148,128 per year, with 24.1% of initiators having an actionable DGI. A previous study estimated the number of drug initiators for 45 drugs with a DPWG recommendation in the Netherlands to be much higher at 3,628,597 new prescriptions per year, with a similar portion of those with actionable DGI (23.6 vs. 24.1%) (Bank et al., 2019). This discrepancy is a result of the reported study using dispersion data from community pharmacies serving primary care. In contrast, our study used data encompassing primary and hospital care. Additionally, the previous study excluded drugs only applied in hospital care such as capecitabine, fluorouracil, and irinotecan. However, similar numbers of drug initiators are reported to be applied both in primary and hospital settings: azathioprine (6,943 vs. 6,979), clopidogrel (98,709 vs. 117,900), mercaptopurine (2,598 vs. 2,177) and thiopurine (1,883 vs. 2,854). Despite a seemingly large discrepancy initially, these numbers confirm the accuracy of the number of yearly drug initiators in the presented model.

In the presented analysis, we limited the input of costs to PGx-testing, HCP interpretation, and drugs and thereby we have excluded the cost of hospitalization as a result of gene-drug-related ADRs which do not lead to death. Despite this limited perspective, we argue that we have been conservative in estimation of costs. For example, the cost of PGx tests were based on 2018 LUMC prices, which are higher than the current prices in 2020. This confirms the prediction that costs of genetic tests are decreasing. Although performed with a different PGx intervention and target population, PGx cost-savings have previously been estimated at $218 per tested patient (Brixner et al., 2016). Additional cost-savings that were excluded are the reduced healthcare utilization resulting from reduced dose switching or reduced clinical monitoring (Toffoli et al., 2019). As a result, we are conservative in the cost of preventing gene-drug-related deaths and underestimate additional cost-saving.

### 4.3 Limitations

A key limitation of our approach is that the selected publications for risk of gene-related death extraction were powered on intermediate outcomes and not on drug-induced mortality (those corresponding to a certainty score 3 and lower). However, we do not expect PGx studies to be powered on mortality since these would require large sample sizes. As a result, we had to resort to the extraction of the absolute risk of intermediary outcomes, such as drug-induced myelosuppression, that are known to be associated with gene-drug-related death and multiplied this with the risk of mortality as a result of this intermediary outcome. While the extraction of the risk of mortality and intermediary outcomes was performed systematically based on literature underlying the DPWG, the risk of death as a result of intermediary outcomes was non-systematic, driven by the investigators’ judgment of being suitable. Additionally, the majority of effect-sizes of PGx-guide prescribing to prevent gene-drug-related deaths are extracted from a number of observational studies. Ideally, these would be extracted from randomized controlled trials (RCTs) directly comparing PGx intervention to standard of care. However, we feel extraction from observational studies is substantiated since we do not expect RCTs to be performed for every individual DGI.

### 4.4 Future Research

The current study reports on seven “essential” DGIs in single-gene scenarios, but many more recommendations for actionable DGIs are available which intend to prevent non-fatal ADRs. From 2005 onwards the DPWG has developed 63 recommendations (Swen et al., 2008; Swen et al., 2011; Swen et al., 2018) and in parallel, the CPIC has devised 73 recommendations (Relling and Klein, 2011; Relling et al., 2020). In the near future, PGx delivery will shift from single-gene reactive model to a pre-emptive panel-testing model. Here, multiple pharmacogenes are tested simultaneously and recorded in the EMR in preparation of future prescriptions. Pre-emptive panel-testing may optimize both logistics and cost-effectiveness. This is supported by the observation that patients will receive multiple drug prescriptions with potential DGIs within their lifetime (Schildcrout et al., 2012; Samwald et al., 2016) and the fact that marginal acquisition costs of testing and interpreting additional pharmacogenes is near-zero (Roden et al., 2018). However, the pre-emptive nature may also reduce cost-effectiveness, as not all tested individuals will actually benefit from the testing. Therefore, as implementation of PGx transitions from a single-gene approach to a pre-emptive panel approach, future efforts should quantify the cost-effectiveness of a panel of pharmacogenes to guide dose and drug selection of the remaining DGIs for which guidelines are available and over a longer time-horizon.

## 5 Conclusion

We used a decision-analytic model to assess the cost-effectiveness of nation-wide PGx-guided initial drug treatment for seven DGIs categorized as “essential” by the DPWG in the Netherlands. We found that nation-wide adoption of PGx-guided initial dose and drug selection of “essential” DGIs can potentially save the lives of 419 (0.3% of drug initiators) at reasonable costs (€51,000 per prevented death). The weighted average certainty score was 2.5 (fairly certain). These results support nation-wide adoption of PGx-guided initial drug treatment for “essential” DGIs.

## Data Availability

The original contributions presented in the study are included in the article/Supplementary Material, further inquiries can be directed to the corresponding author.
