# Supplementary material for: Cost-Effectiveness of Pharmacogenomics-Guided Prescribing to Prevent Gene-Drug-Related Deaths: A Decision-Analytic Model
Source: Front Pharmacol. 2022 Jun 28;13:918493. doi: 10.3389/fphar.2022.918493 (PMC9477094; doi:10.3389/fphar.2022.918493)
Supplement: Supplementary file 1 [file DataSheet1.pdf]

Supplementary Material

Supplementary Table 1: Overview of the used initiator/user ratios and calculated number of drug initiators

Drug	Number of nation-wide users from GIP databank	N initiators in LUMC in 2018	N users + N initiators and users in LUMC in 2018	Ratio initiators/users	Calculated N drug initiators nation-wide
Azathioprine	26,153	317	1,188	0.267	26,153
Capecitabine	11,966	194	262	0.740	11,966
Clopidogrel	315,877	2,447	6,556	0.373	315,877
Fluorouracil (systemic + cutaneous)	54,815	442	1,110	-	-
Fluorouracil (cutaneous)	-	305	944	-	-
Fluorouracil (systemic)	8,198*	137	166	0.825	8,198
Irinotecan	2,593	48	0	1	2,593
Mercaptopurine	6,411	36	106	0.340	6,411
Tioguanine	5,116	82	147	0.558	5,116

*Calculated by multiplying with % systemic users in LUMC in 2018 (= N systemic users/ N systemic + N cutaneous users in 2018 = 166/1,110*100%=14.95%)

Supplementary Table 2 Systematic methodology to select publications and extract absolute risk of gene-drug-related-death

The steps shown in **Table 1** are performed systematically to select relevant publications from which to extract the absolute risk of gene-drug-related death. Risk extraction is performed by using methodology corresponding to that step. Each extracted absolute risk of death is given a certainty score based on the step in which publication(s) are selected.

The publication selection is performed systematically using only the publications listed in the summary of the systematic review of literature underlying the DPWG guideline ("the risk analysis"). Each of the publications listed in the risk analysis have been scored systematically by the DPWG both on the clinical relevance and on the quality of evidence [1]. The quality of evidence for each publication was scored on a five-point scale ranging from 0 (lowest quality of evidence) to 4 (highest quality of evidence). Score 4 corresponds to controlled, published studies of good quality or well-performed meta-analyses. Good quality is defined as: it is known whether comedication with an influence on the phenotype has been used; it is known whether other confounders are present (depending on the substance, for example smoking or not); the data are based on steady state kinetics; corrected for this at a variable dose [2]. Score 3 corresponds to controlled, published studies of moderate quality or poorly performed meta-analyses (for example, no good statistics, studies with different measured endpoints, heterogeneity, publication bias). Moderate quality is defined as: at least one of the criteria considered under good quality does not apply [2].

The risk of gene-drug-related death will vary across predicted phenotype groups. For example, risk of fluoropyrimidine-induced toxicity increases with decreasing DPYD gene activity scores (GAS), when all groups receive the same initial dose. Furthermore, when a PGx test is used to guide dose selection, those who have an actionable predicted phenotype (DPYD GAS 0-1.5) will have a reduced risk of fluoropyrimidine-induced toxicity when compared to risk when using a normal dose. The risk of death as a result of fluoropyrimidine-induced toxicity, however, in those with a non-actionable predicted phenotype (in this case DPYD GAS 2) will have the same risk, regardless of being PGx tested. Therefore, we will extract the absolute risk of death for each predicted phenotype category, across three groups: 1) tested-actionables (e.g. DPYD GAS 0, 0.5, 1 and 1.5 with PGx informed reduced dose), 2) non-actionables (e.g. DPYD GAS 2 with normal dose) and 3) untested-actionables (e.g. DPYD GAS 0, 0.5, 1 and 1.5 with normal dose). The predicted phenotype-gene interactions which are categorized as being actionable or non-actionable are provided in Table 1.

Other publications may be selected for extraction of each absolute risk. For example, risks of untested-actionables and non-actionables groups may be extracted from observational studies. However, the risks of tested-actionables group must be extracted from interventional studies. When a publication is selected for one of these three groups within one step, but is not suitable for risk extraction of the remaining groups, the following step is performed to find a suitable publication for the remaining groups.

Table 1: Systematic methodology to select suitable publications and subsequent extraction of absolute risk of gene-drug-related-death within one year. The steps are executed consecutively until at least one suitable publication is found.

**Step**	**Suitable publication(s)**	**Risk extraction method**	**1) tested actionables 2) non-actionables 3) untested actionables**	**Certainty Score**
1	Publications reporting predicted phenotype group: quality score 4[a], powered on mortality	The risk of mortality of the most severe preventable clinical consequence within one year is extracted.		4 = Very certain
2	Publications reporting predicted phenotype group: quality score 4[a]	The risk of the intermediary outcome within one year is extracted and is multiplied by the risk of death as a result of this intermediary outcome within one year. This is found by searching literature.		3 = Certain
3	Publications reporting predicted phenotype group: quality score 3[b], powered on mortality	The risk of mortality of the most severe preventable clinical consequence within one year is extracted.		2 = Fairly certain
4	Publications reporting predicted phenotype group: quality score 3[b]	The risk of the intermediary outcome within one year is extracted and is multiplied by the risk of death as a result of this intermediary outcome within one year. This is found by searching literature.		1 = Uncertain
5	Perform literature review in review of a usable study regarding the relevant DGI	When the study is powered on mortality the risk of mortality within one year is extracted. When the study reported on an intermediary outcome, the intermediary outcome within one year is extracted and is multiplied by the risk of death as a result of this intermediary outcome within one year. This is found by searching literature.		Based on quality score criteria of DPWG
6 No publication selected	Estimation			0 = Very uncertain

[a] Controlled, published studies of good quality with genotyping and / or phenotyping in patients / healthy subjects with clinical endpoints (effectiveness, side effects) or relevant kinetic endpoints (change in plasma level, AUC, half-life, etc.) or good performed meta-analyzes. Good quality is defined as: it is known whether comedication with an influence on the phenotype has been used; it is known whether other confounders are present (depending on the substance, for example smoking or not); the data are based on steady state kinetics; corrected for this at a variable dose [2]. [b] Controlled, published studies of moderate quality. with genotyping and / or phenotyping in patients / healthy subjects with clinical endpoints (effectiveness, side effects) or relevant kinetic endpoints (change in

plasma level, AUC, half-life, etc.) or poor performed meta-analyzes (for example, no good statistics, studies with different measured endpoints, heterogeneity, publication bias). Moderate quality means that one or more of the items considered under good quality are missing [2].

Publication(s) selection

Publications are selected only if they present usable risk data and are sufficiently representative for the healthcare system and patients in the Netherlands. Being usable is defined as presenting risk data from which risks for at least one of the three groups can be calculated without requesting raw data underlying the publication. Being sufficiently representative is defined as studies including patients of which at least 50% are from North America or Europe.

Absolute risk extraction

Once at least one publication has been selected for each relevant drug-phenotype category for three patient groups: 1) tested-actionables, 2) non-actionables and 3) untested-actionables we are able to extract risks. This is performed corresponding to the step in which the publication was selected (see below).

Within a particular step, if only one publication is selected, the absolute risks of death are extracted from that single publication. When more than one publications are found suitable, the absolute risks of death are extracted from each publication and the mean is taken (weighed by the number of patients). However, when multiple meta-analyses are selected within one step, the risk extraction will only be performed based on the most recent meta-analysis, provided the majority of studies included in older meta-analyses.

Step 1: Publications reporting predicted phenotype group: quality score 4, powered on mortality (certainty score 4)

The risk of mortality of the most severe preventable clinical consequence within one year is extracted directly.

Step 2: Publications reporting predicted phenotype group: quality score 4, calculating the risk of death from intermediary outcome (certainty score 3)

The risk of an intermediary outcome within one year is extracted and is multiplied by the risk of death as a result of this intermediary outcome within one year. Risk of death as a result of an intermediary outcome is found by searching literature and presented in Appendix 2 section "Assessment of risk of drug-related death following an intermediary outcome associated with the gene-drug interaction".

Step 3: Publications reporting predicted phenotype group: quality score 3, powered on mortality (certainty score 2)

The risk of mortality of the most severe preventable clinical consequence within one year is extracted.

Step 4: Publications reporting predicted phenotype group: quality score 3, calculating the risk of death from intermediary outcome (certainty score 1)

The risk of the intermediary outcome within one year is extracted and is multiplied by the risk of death as a result of this intermediary outcome within one year. Risk of death as a result of an intermediary outcome is found by searching literature and presented in Appendix 2 section "Assessment of risk of drug-related death following an intermediary outcome associated with the gene-drug interaction".

Step 5: Perform literature review in review of a usable study regarding the relevant DGI (certainty score is based on quality of evidence criteria of DPWG)

When the study is powered on mortality the risk of mortality within one year is extracted. When the study reported on an intermediary outcome, the intermediary outcome within one year is extracted and is multiplied by the risk of death as a result of this intermediary outcome within one year. This is found

by searching literature and presented in Appendix 2 section "Assessment of risk of drug-related death following an intermediary outcome associated with the gene-drug interaction".

Step 6: No publication selected: (certainty score 0 - estimation)

When none of the selected publications are intervention studies, we are unable to extract the risk of death for tested actionables. In this case we estimate the risk of death for tested actionables to equal the risk of death of non-actionables. In this case it is given a certainty score of 0 (estimation).

References:

1. Swen JJ, Wilting I, de Goede AL, Grandia L, Mulder H, Touw DJ, et al. Pharmacogenetics: from bench to byte. Clinical pharmacology and therapeutics. 2008;83(5):781-7.
2. https://kennisbank.knmp.nl/files/farmacogenetica/Achtergrondteksten/fgbk.pdf

Supplementary Table 3 Systematic selection of literature and extraction of absolute risk of gene-drug-related death

1.1 *TPMT*-AZATHIPURINE/MERCAPTOPURINE 7
1.1.1 Publication selection 7
1.1.2 Absolute risk extraction non-actionables (EM): 12
1.1.3 Absolute risk extraction untested actionables (IM and PM): 13
1.1.4 Absolute risk extraction tested actionables (IM and PM): 13
1.1.5 Conclusion of selected publications and absolute risks extracted: 14
1.2 *TPMT*-TIOGUANINE 15
1.2.1 Publication selection 15
1.2.2 Conclusion of selected publications and absolute risks extracted: 16
1.3 *DPYD*-CAPECITABINE/5-FU 17
1.3.1 Publication selection 17
1.3.2 Absolute risk extraction non-actionables (GAS 2): 22
1.3.3 Absolute risk extraction untested actionables (GAS 0-1.5): 23
1.3.4 Absolute risk extraction tested actionables (GAS 0-1.5): 24
1.3.5 Conclusion of selected publications and absolute risks extracted: 27
1.4 *CYP2C19*-CLOPIDOGREL 28
1.4.1 Publication selection 28
1.4.2 Absolute risk extraction non-actionables (UM and EM): 35
1.4.3 Absolute risk extraction untested actionables (IM and PM): 35
1.4.4 Absolute risk extraction tested actionables (IM and PM): 35
1.4.5 Conclusion of selected publications and absolute risks extracted: 36
1.5 *UGT1A1*-IRINOTECAN 38
1.5.1 Publication selection 38
1.5.2 Absolute risk extraction non-actionables (*1/*1, *1/*28 and IM): 44
1.5.3 Absolute risk extraction untested actionables (*28/*28 and PM): 44
1.5.4 Absolute risk extraction tested actionables (*28/*28 and PM): 45
1.5.5 Conclusion of selected publications and absolute risks extracted (death as a result of neutropenia): 45
1.5.6 Conclusion of selected publications and absolute risks extracted (death as a result of diarrhoea): 45

1.5.7 Conclusion of selected publications and absolute risks extracted (sum absolute risk of death due to neutropenia and absolute risk of death due to diarrhoea): 46
1.6 Assessment of risk of drug-related death following an intermediary outcome associated with the gene-drug interaction 47
1.7 References 49

1.1 *TPMT*-AZATHIPURINE/MERCAPTOPURINE

1.1.1 Publication selection

Risk analysis: https://kennisbank.knmp.nl/files/farmacogenetica/1905-1906.PDF

Since the risk analysis is combined for both azathioprine and mercaptopurine, the publication selection and risk extraction will also be combined for both.

There is a DPWG guideline for the indications of acute lymphoblastic leukaemia (ALL) and irritable bowel syndrome (IBD). We have chosen to only select literature for application of *TPMT* guided prescribing for IBD. Reason for this being that the majority of patients initiating thiopurines have an IBD indication.

<table>
<tr><td></td><td>Steps performed systematically to select suitable publication(s) form which extraction is performed</td><td>Publication(s) selection</td></tr>
<tr><td>1</td><td>Publications reporting predicted phenotype group: quality score 4, powered on mortality</td><td>
<table>
<tr><th>Study</th><th>Conclusion</th></tr>
<tr><td>Relling MJ et al.(1)</td><td>Not powered on mortality. Not selected.</td></tr>
<tr><td>Lui C et al.(2)</td><td>Not powered on mortality. Not selected.</td></tr>
<tr><td>Booth RA et al.(3)</td><td>Not powered on mortality. Not selected.</td></tr>
<tr><td>Zelinkova Z et al.(4)</td><td>Not powered on mortality. Not selected.</td></tr>
<tr><td>Fabre MA et al. (5)</td><td>Not powered on mortality. Not selected.</td></tr>
<tr><td>Pandya B et al.(6)</td><td>Not powered on mortality. Not selected.</td></tr>
<tr><td>Stanulla M et al.(7)</td><td>Not powered on mortality. Not selected.</td></tr>
</table>
Conclusion:
No literature was selected therefore we will continue to the next step.</td></tr>
<tr><td>2</td><td>Publications reporting predicted</td><td>
<table>
<tr><th>Study</th><th>Conclusion</th></tr>
</table></td></tr>
</table>

	phenotype group: quality score 4	Relling MJ et al.(1)	Usable risk data: Yes<br>Representative: No, indication in this study is ALL patients.<br>Not selected.
		Lui C et al.(2)	Representative: No, indication in this study is ALL patients.<br>Not selected.
		Booth RA et al.(3)	Usable risk data: Yes<br>Representative: Yes<br><br>**Selected for extraction of untested and non-actionable groups.**
		Zelinkova Z et al.(4)	Representative: Yes<br>Usable risk data: Yes<br>Not selected. (Data is included in included meta-analysis by Booth et al.)
		Fabre MA et al. (5)	Representative: No, indication in kidney transplantation patients.<br>Not selected.
		Pandya B et al.(6)	Representative: No, indication in kidney transplantation patients.<br>Not selected.
		Stanulla M et al.(7)	Representative: No, indication in this study is ALL patients.<br>Not selected.
		Conclusion:<br>Booth RA et al.(3) was selected for extraction of untested and non-actionable groups. Therefore we will continue with the next step to obtain the data for the tested groups.	
3	Publications reporting predicted phenotype group: quality score 3, powered on mortality	**Study**	**Conclusion**
		Fan X et al. (8)	Not powered on mortality. Not selected.
		Choi R et al. (9)	Not powered on mortality. Not selected.
		Eriksen P et al. (10)	Not powered on mortality. Not selected.
		Coenen MJ et al. (11)	Not powered on mortality. Not selected.
		Lennard L et al. (12)	Not powered on mortality. Not selected.

			Lennard L et al. (13)	Not powered on mortality. Not selected.	
			Kim MJ et al. (14)	Not powered on mortality. Not selected.	
			Leninsen M et al. (15)	Not powered on mortality. Not selected.	
			Kim H et al. (16)	Not powered on mortality. Not selected.	
			Newman W et al. (17)	Not powered on mortality. Not selected.	
			Dong XW et al. (18)	Not powered on mortality. Not selected.	
			Hildorf U et al. (19)	Not powered on mortality. Not selected.	
			Sheffiled L et al. (20)	Not powered on mortality. Not selected.	
			Ansari A et al. (21)	Not powered on mortality. Not selected.	
			Gardiner S et al. (22)	Not powered on mortality. Not selected.	
			Moloney FJ et al. (23)	Not powered on mortality. Not selected.	
			Jun JB et al. (24)	Not powered on mortality. Not selected.	
			Stocco G et al. (25)	Not powered on mortality. Not selected.	
			Kurzawski M et al. (26)	Not powered on mortality. Not selected.	
			Gearry RB et al. (27)	Not powered on mortality. Not selected.	
			Ansari A et al. (28)	Not powered on mortality. Not selected.	
			Langley P et al. (29)	Not powered on mortality. Not selected.	
			Regueiro M et al. (30)	Not powered on mortality. Not selected.	
			Campbell S et al. (31)	Not powered on mortality. Not selected.	
			Colombel JF et al. (32)	Not powered on mortality. Not selected.	
			Black AJ et al. (33)	Not powered on mortality. Not selected.	
			Higgs JE et al. (34)	Not powered on mortality. Not selected.	

		Evans et al. (35)	Not powered on mortality. Not selected.
		McLeod HL et al. (36)	Not powered on mortality. Not selected.
		Conclusion:<br>No literature was selected therefore we will continue to the next step.	
4	Publications reporting predicted phenotype group: quality score 3	**Study**	**Included in input data**
		Fan X et al. (8)	Representative: No, Chinese patients.<br><br>Not selected.
		Choi R et al. (9)	Representative: No, Korean pediatric ALL patients.<br><br>Not selected.
		Eriksen P et al. (10)	Representative: No, autoimmune hepatitis patients.<br><br>Not selected.
		Coenen MJ et al. (11)	Representative: Yes<br><br>Usable risk data: Yes<br>**Selected for extraction of tested groups.**
		Lennard L et al. (12)	Representative: No, ALL patients.<br><br>Not selected.
		Lennard L et al. (13)	Representative: No, ALL patients.<br><br>Not selected.
		Kim MJ et al. (14)	Representative: No, Korean patients.<br><br>Not selected.
		Leninsen M et al. (15)	Representative: No, ALL patients.<br><br>Not selected.
		Kim H et al. (16)	Representative: No, ALL patients.<br><br>Not selected.
		Newman W et al. (17)	Representative: Yes<br>Usable risk data: No

	Not selected.
Dong XW et al. (18)	Representative: No, less <50% of studies western.<br>Not selected.
Hildorf U et al. (19)	Representative: No, autoimmune hepatitis patients.<br><br>Not selected.
Sheffiled L et al. (20)	Representative: Yes<br>Usable risk data: No, not genotype-guided.<br>Not selected.
Ansari A et al. (21)	Representative: Yes<br><br>Usable risk data: No, not genotype-guided.<br><br>Not selected.
Gardiner S et al. (22)	Representative: Yes<br>Usable risk data: No, not genotype-guided.<br><br>Not selected.
Moloney FJ et al. (23)	Representative: No, renal transplant patients.<br><br>Not selected.
Jun JB et al. (24)	Representative: No, lupus erythematosus patients.<br><br>Not selected.
Stocco G et al. (25)	Representative: No, only pediatric patients.<br>Usable risk data: No, not genotype-guided.<br>Not selected.
Kurzawski M et al. (26)	Representative: No, renal transplant patients.<br><br>Not selected.
Gearry RB et al. (27)	Representative: Yes<br><br>Usable risk data: No, not genotype-guided.<br>Not selected.
Ansari A et al. (28)	Representative: Yes<br><br>Usable risk data: No, not genotype-guided.<br>Not selected.

<table>
<tr>
<td></td>
<td></td>
<td>
<table>
<tr><td>Langley P et al. (29)</td><td>Representative: No, autoimmune hepatitis patients.

Not selected.</td></tr>
<tr><td>Regueiro M et al. (30)</td><td>Representative: Yes

Usable risk data: No
Not selected.</td></tr>
<tr><td>Campbell S et al. (31)</td><td>Representative: Yes

Usable risk data: No, not genotype-guided.
Not selected.</td></tr>
<tr><td>Colombel JF et al. (32)</td><td>Representative: Yes

Usable risk data: No, not genotype-guided.
Not selected.</td></tr>
<tr><td>Black AJ et al. (33)</td><td>Representative: No, rheumatic patients.
Not selected.</td></tr>
<tr><td>Higgs JE et al. (34)</td><td>Representative: No, not specific for IBD.

Usable risk data: No, not genotype-guided.
Not selected.</td></tr>
<tr><td>Evans et al. (35)</td><td>Representative: No, ALL patients.

Not selected.</td></tr>
<tr><td>McLeod HL et al. (36)</td><td>Representative: No, ALL patients.

Not selected.</td></tr>
</table>
Conclusion:
Coenen MJ et al. (11) was selected for extraction for tested-actionable groups.
</td>
</tr>
<tr>
<td>5</td>
<td>Perform literature review in review of a usable study regarding the relevant DGI</td>
<td>Not applicable</td>
</tr>
</table>

1.1.2 Absolute risk extraction non-actionables (EM):

Booth RA et al.(3) was selected for extraction of non-actionables groups.
Booth RA et al.(3) is a meta-analysis of 31 studies into toxicity caused by azathioprine or mercaptopurine in a total of 3,638 patients with autoimmune diseases (including 260 IM and 19 PM). Leukopenia was the measure of outcome in 18 studies involving a total of 1,825 patients, including 105 IM and 7 PM.

Risk of leukopenia was 0.209573847 (See Appendix Figure 2, sum of events/sum of patients = 359/1713) among non-actionable TPMT EMs. Risk of death among IBD patients who develop myelotoxicity is approximately 0.01 (37). Therefore, risk of death as a result of leukopenia is 0.209573847 x 0.01 = 0.002095738 for non-actionable TPMT EMs. These are given a certainty score of 3.

1.1.3 Absolute risk extraction untested actionables (IM and PM):

Booth RA et al.(3) was selected for extraction of untested-actionables groups.
Booth RA et al. (3) is a meta-analysis of 31 studies into toxicity caused by azathioprine or mercaptopurine in a total of 3,638 patients with autoimmune diseases (including 260 IM and 19 PM). Leukopenia was the measure of outcome in 18 studies involving a total of 1,825 patients, including 105 IM and 7 PM.

TPMT IM:
Risk of leukopenia was 0.39047619 (See Appendix Figure 2, sum of events/sum of patients = 41/105) among untested-actionable TPMT IMs. Risk of death among IBD patients who develop myelotoxicity is approximately 0.01(37). Therefore, risk of death as a result of leukopenia is 0.39047619 x 0.01 = 0.003904762 for untested TPMT IMs. These are given a certainty score of 3.
TPMT PM:
The absolute number of leukopenia events is not presented for PMs. However, paragraph Enzyme Activity notes that the odds of leukopenia were significantly greater with low TPMT activity than with intermediate (OR= 2.74 [CI, 1.54 to 4.86]; 4 studies, 257 patients, and 91 events). Therefore the risk of leukopenia was calculated to be [untested-actionable TPMT IM = 0.39047619] X [OR of 2.74] = 1.069904 = 1. Risk of death among IBD patients who develop myelotoxicity is approximately 0.01(37). Therefore, risk of death as a result of leukopenia is 1 x 0.01 = 0.01 for untested TPMT PMs. These are given a certainty score of 3.

1.1.4 Absolute risk extraction tested actionables (IM and PM):

Coenen MJ et al. (11) was selected for extraction for tested-actionable groups. Coenen MJ et al. (11) is a randomized controlled trial. Here, 783 patients with IBD were treated with azathioprine (64% of patients) or 6-mercaptopurine (36% of patients). Follow-up was for a period of 20 weeks. Genotype-guided (*TPMT* *2, *3A and *3C) treatment (n = 405) was compared to standard treatment (n = 378). In the genotype-guided group, EMs received the normal thiopurine dose and IMs 50% of the normal dose. PM were scheduled to receive 0-10% of the normal dose. Hematologic adverse events were defined as leukocyte count < 3.0x109/L or platelet count < 100x109/L.A significantly smaller proportion of carriers of the TPMT variants in the intervention group (2.6%) developed hematologic ADRs compared with patients in the control group (22.9%) (relative risk, 0.11; 95% confidence interval, 0.01-0.85).
TPMT IM and PM:
Coenen et al. has combined the *TPMT* IMs and PMs in one group, therefore we will also perform risk extraction for IM and PMs combined. Risk of hematologic adverse events was 0.025641026 among tested TPMT IMs and PMs (1 event among 39 patients, see Table 3). Risk of death among IBD patients who develop myelotoxicity is approximately 0.01 (37). Therefore, risk of death as a result of leukopenia is 0.025641026 x 0.01 = 0.025641026 for tested TPMT IMs and PMs. These are given a certainty score of 1.

1.1.5 Conclusion of selected publications and absolute risks extracted:

		Actionability	Untested	Ref	CS	Tested	Ref	CS
Azathiopurine/ Mercaptopurine	TPMT EM	no	0.002095738	(3)	3	0.002095738	(3)	3
Azathiopurine/ Mercaptopurine	TPMT IM	yes	0.003904762	(3)	3	0.00025641	(11)	1
Azathiopurine/ Mercaptopurine	TPMT PM	yes	0.01	(3)	3	0.00025641	(11)	1

Ref: Reference; CS: Certainty score

1.2 *TPMT*-TIOGUANINE

1.2.1 Publication selection

Risk analysis: https://kennisbank.knmp.nl/files/farmacogenetica/1907-1908.PDF

<table>
<tr><td></td><td>Steps performed systematically to select suitable publication(s) form which extraction is performed</td><td>Publication(s) selection</td></tr>
<tr><td>1</td><td>Publications reporting predicted phenotype group: quality score 4, powered on mortality</td><td>There are no studies available through the “risk analysis” that have a quality score of 4.
Conclusion:
No literature was selected therefore we will continue to the next step.</td></tr>
<tr><td>2</td><td>Publications reporting predicted phenotype group: quality score 4</td><td>There are no studies available through the “risk analysis” that have a quality score of 4.
Conclusion:
No literature was selected therefore we will continue to the next step.</td></tr>
<tr><td>3</td><td>Publications reporting predicted phenotype group: quality score 3, powered on mortality</td><td><table>
<tr><th>Study</th><th>Conclusion</th></tr>
<tr><td>Lennard L et al. (13)</td><td>Not powered for mortality</td></tr>
<tr><td>Wray L et al. (38)</td><td>Not powered for mortality</td></tr>
<tr><td>Lennard L et al. (39)</td><td>Not powered for mortality</td></tr>
<tr><td>Teml A et al. (40)</td><td>Not powered for mortality</td></tr>
<tr><td>Herrlinger KR et al.(41)</td><td>Not powered for mortality</td></tr>
</table>Conclusion:
No literature was selected therefore we will continue to the next step.</td></tr>
<tr><td>4</td><td>Publications reporting predicted phenotype group: quality score 3</td><td><table>
<tr><th>Study</th><th>Conclusion</th></tr>
<tr><td>Lennard L et al. (13)</td><td>Usable risk data: No</td></tr>
<tr><td>Wray L et al. (38)</td><td>Usable risk data: No
Patients are children with ALL.</td></tr>
<tr><td>Lennard L et al. (39)</td><td>Usable risk data: No
Patients are children with ALL.</td></tr>
<tr><td>Teml A et al. (40)</td><td>Usable risk data: No. Very small study population.</td></tr>
</table></td></tr>
</table>

		Herrlinger KR et al.(41) — Usable risk data: No. Very small study population.<br>Conclusion:<br>No literature was selected therefore we will continue to the next step.
5	Perform literature review in review of a usable study regarding the relevant DGI	**Search strategy pubmed** — **Date literature search**<br>(Thioguanine[Title] OR Tioguanine[Title] OR 6-thioguanine[Title] OR 6-TG[Title]) AND (TPMT[Title] OR Thiopurine[Title] OR Pharmacogenetic[Title] OR Pharmacogenetics [Title] OR genotype[Title] OR genotypes[Title] OR polymorphism[Title] OR polymorphisms[Title]) — 02-12-2019<br>Conclusion:<br>We found no additional studies through our own literature search. Therefore, we estimated the absolute risk on death for thioguanine to be similar to azathioprine and 6-mercaptopurine. The certainty score given is 0, since it is an estimation.

1.2.2 Conclusion of selected publications and absolute risks extracted:

		Actionability	Untested	Ref	CS	Tested	Ref	CS
Thioguanine	TPMT EM	no	0.002095738	(3)	0	0.002095738	(3)	0
Thioguanine	TPMT IM	yes	0.003904762	(3)	0	0.00025641	(11)	0
Thioguanine	TPMT PM	yes	0.01	(3)	0	0.00025641	(11)	0

1.3 *DPYD*-CAPECITABINE/5-FU

1.3.1 Publication selection

Risk analysis: https://kennisbank.knmp.nl/files/farmacogenetica/2552-4893-4894.PDF

Since the risk analysis is combined for both capecitabine and 5-FU, the publication selection and risk extraction will also be combined for both.

	Steps performed systematically to select suitable publication(s) form which extraction is performed	Publication(s) selection
1	Publications reporting predicted phenotype group: quality score 4, powered on mortality	**Study** / **Conclusion**<br>Deenen MJ et al. (42): Not powered on mortality. Not selected.<br>Meulendijks D et al. (43): Not powered on mortality. Not selected.<br>Meulendijks D et al.(44): Not powered on mortality. Not selected.<br>Rosmarin D et al.(45): Not powered on mortality. Not selected.<br>Terrazzino S et al. (46): Not powered on mortality. Not selected.<br>Vulsteke C et al. (47): Not powered on mortality. Not selected.<br>Conclusion:<br>No publication was selected therefore we will continue to the next step.
2	Publications reporting predicted phenotype group: quality score 4	**Study** / **Conclusion**<br>Deenen MJ et al. (42): Usable risk data: Yes (includes alleles relevant for Dutch population)<br>Representative: Yes<br>Not selected. (Study is present in the included meta-analysis by Meulendijks et al. (44))<br>Meulendijks D et al. (43): Usable risk data: Yes<br>Representative: No. Study only in small population for specific alleles.<br>Not selected.

		Meulendijks D et al.(44)	Usable risk data: Yes<br>Representative: Yes<br>**Selected for extraction of untested groups.**
		Rosmarin D et al.(45)	Usable risk data: No<br><br>Representative: Yes<br>Not selected.
		Terrazzino S et al. (46)	Usable risk data: Yes<br>Representative: Yes<br>Not selected. Another meta-analysis is more recent.
		Vulsteke C et al. (47)	Usable risk data: Yes.<br>Representative: Yes<br><br>Not selected. Another meta-analysis is more recent.
		Conclusion:<br>Meulendijks D et al.(44) was selected for extraction of untested and non-actionable groups. Therefore we will continue with the next step to obtain the data for the tested groups..	
3	Publications reporting predicted phenotype group: quality score 3, powered on mortality	**Study**	**Conclusion**
		Kleinjan JP et al.(48)	Not powered on mortality. Not selected.
		Henricks LM et al. (49)	Not powered on mortality. Not selected.
		Lunenburg CATC et al. (50)	Not powered on mortality. Not selected.
		Henricks LM et al.(51)	Not powered on mortality. Not selected.
		Madi A et al. (52)	Not powered on mortality. Not selected.
		Lunenburg CA et al.(53)	Not powered on mortality. Not selected.
		Lee AM et al. (54)	Not powered on mortality. Not selected.
		Deenen MJ et al. (55)	Not powered on mortality. Not selected.

		Lee AM et al. (56)	Not powered on mortality. Not selected.	
		van Kuilenburg AB et al.(57)	Not powered on mortality. Not selected.	
		Kristensen MH et al.(58)	Not powered on mortality. Not selected.	
		Gross E et al.(59)	Not powered on mortality. Not selected.	
		Capitain O et al. (60)	Not powered on mortality. Not selected.	
		Sulzyc-Bielicka V et al.(61)	Not powered on mortality. Not selected.	
		Schwab M et al. (62)	Not powered on mortality. Not selected.	
		Mercier C et al.	Not powered on mortality. Not selected.	
		Jatoi A et al.(63)	Not powered on mortality. Not selected.	
		Magné N et al. (64)	Not powered on mortality. Not selected.	
		Boisdron-Celle M et al. (65)	Not powered on mortality. Not selected.	
		Cho HJ et al. (66)	Not powered on mortality. Not selected.	
		Salgado J et al. (67)	Not powered on mortality. Not selected.	
		Morel A et al. (68)	Not powered on mortality. Not selected.	
		Largillier R et al. (69)	Not powered on mortality. Not selected.	
		Salgueiro N et al. (70)	Not powered on mortality. Not selected.	
		Van Kuilenburg AB et al.(71)	Not powered on mortality. Not selected.	
		Raida M et al. (72)	Not powered on mortality. Not selected.	
		Yamaguchi K et al. (73)	Not powered on mortality. Not selected.	

		van Kuilenburg AB et al.(74)	Not powered on mortality. Not selected.
		Conclusion:<br>No publications were selected therefore we will continue to the next step.	
4	Publications reporting predicted phenotype group: quality score 3	**Study**	**Conclusion**
		Kleinjan JP et al.(48)	Representative: Yes, Dutch population.<br><br>Usable risk data: Yes<br>**Selected for extraction of tested groups.**
		Henricks LM et al. (49)	Representative: Yes, Dutch population.<br><br>Usable risk data: Yes<br>**Selected for extraction of tested groups.**
		Lunenburg CATC et al. (50)	Representative: Yes, Dutch population.<br><br>Usable risk data: Yes<br>**Selected for extraction of tested groups.**
		Henricks LM et al.(51)	Representative: Yes<br>Usable risk data: Yes<br>**Selected for extraction of tested groups.**
		Madi A et al. (52)	Usable risk data: no risk for tested actionables reported.<br>Not selected.
		Lunenburg CA et al.(53)	Representative: Yes , Dutch population.<br>Usable risk data: No<br><br>Not selected.
		Lee AM et al. (54)	Usable risk data: no risk for tested actionables reported.<br>Not selected.
		Deenen MJ et al. (55)	Representative: Yes, Dutch population.<br>Usable risk data: Yes<br><br>**Selected for extraction of tested groups?**

			Lee AM et al. (56)	Usable risk data: no risk for tested actionables reported.<br>Not selected.	
			van Kuilenburg AB et al.(57)	Representative: Yes, Dutch population.<br>Usable risk data: No, not genotype-guided.<br>Not selected.	
			Kristensen MH et al.(58)	Usable risk data: no, not genotype-guided.<br>Not selected.	
			Gross E et al.(59)	Usable risk data: No, not genotype-guided.<br>Not selected.	
			Capitain O et al. (60)	Usable risk data: No, not genotype-guided.<br>Not selected.	
			Sulzyc-Bielicka V et al.(61)	Usable risk data: No, not genotype-guided.<br>Not selected.	
			Schwab M et al. (62)	Usable risk data: No, not genotype-guided.<br>Not selected.	
			Mercier C et al.	Representative: Yes<br>Usable risk data: No<br>Not selected.	
			Jatoi A et al.(63)	Representative: Yes<br>Usable risk data: No<br>Not selected.	
			Magné N et al. (64)	Representative: Yes<br>Usable risk data: No<br>Not selected.	
			Boisdron-Celle M et al. (65)	Usable risk data: No, not genotype-guided.<br>Representative: Yes<br><br>Not selected.	
			Cho HJ et al. (66)	Representative: No. Study is done in Korean population.<br>Not selected.	

<table>
<tr>
<td></td>
<td></td>
<td>
<table>
<tr><td>Salgado J et al. (67)</td><td>Representative: Yes
Usable risk data: No, not genotype-guided.
Not selected.</td></tr>
<tr><td>Morel A et al. (68)</td><td>Representative: Yes
Usable risk data: No, not genotype-guided.
Not selected.</td></tr>
<tr><td>Largillier R et al. (69)</td><td>Representative: Yes
Usable risk data: No, not genotype-guided.
Not selected.</td></tr>
<tr><td>Salgueiro N et al. (70)</td><td>Representative: Yes
Usable risk data: No, not genotype-guided.
Not selected.</td></tr>
<tr><td>Van Kuilenburg AB et al.(71)</td><td>Representative: Yes
Usable risk data: No, not genotype-guided.
Not selected.</td></tr>
<tr><td>Raida M et al. (72)</td><td>Representative: Yes
Usable risk data: No, not genotype-guided.
Not selected.</td></tr>
<tr><td>Yamaguchi K et al. (73)</td><td>Representative: No. Study is done in Japanese population.
Not selected.</td></tr>
<tr><td>van Kuilenburg AB et al.(74)</td><td>Representative: Yes
Usable risk data: No, not genotype-guided.
Not selected.</td></tr>
</table>
Conclusion: We have selected 5 studies to extract the data for the tested groups:
Kleinjan JP et al.(48)
Henricks LM et al.(49)
Lunenburg CATC et al. (50)
Henricks LM et al.(51)
Deenen M et al.(55)
</td>
</tr>
<tr>
<td>5</td>
<td>Perform literature review in review of a usable study regarding the relevant DGI</td>
<td>Not applicable</td>
</tr>
</table>

1.3.2 Absolute risk extraction non-actionables (GAS 2):

Meulendijks D et al.(44) was selected for extraction of non-actionables groups.

Meulendijks D et al.(44) is a meta-analysis of 8 cohort studies with in total 7365 patients treated with 5-fluorouracil or capecitabine, either as combined chemotherapy (different combinations) or as monotherapy (with or without radiotherapy). Data on *13 were derived from 5 studies including a total of 5,616 patients and 11 carriers of *13. Data on 1236G>A were derived from 6 studies including a total of 4,261 patients and 174 heterozygous carriers and 3 homozygous carriers of 1236A. Data on *2A were derived from 7 studies including a total of 5.737 patients and 60 carriers of *2A. Data on 2846 A>T were derived from all 8 studies including a total of 7,318 patients and 85 carriers of 2846T.

Risk of grade 3 or higher fluoropyrimidine induced toxicity was 0.324008855 (See Figure 2, sum of events/sum of patients =6440/19876) among non-actionable DPYD GAS 2.0. Risk of death as a result of grade 3 or higher fluoropyrimidine induced toxicity is approximately 0.0075 (75). Therefore, risk of death as a result of leukopenia is 0.324008855 x 0.0075 = 0.002430066 for non-actionable DPYD GAS 2.0. These are given a certainty score of 3.

1.3.3 Absolute risk extraction untested actionables (GAS 0-1.5):

Meulendijks D et al.(44). was selected for extraction of untested-actionable groups.
Meulendijks D et al.(44) is a meta-analysis of 8 cohort studies with in total 7365 patients treated with 5-fluorouracil or capecitabine, either as combined chemotherapy (different combinations) or as monotherapy (with or without radiotherapy). Data on *13 were derived from 5 studies including a total of 5,616 patients and 11 carriers of *13. Data on 1236G>A were derived from 6 studies including a total of 4,261 patients and 174 heterozygous carriers and 3 homozygous carriers of 1236A. Data on *2A were derived from 7 studies including a total of 5.737 patients and 60 carriers of *2A. Data on 2846 A>T were derived from all 8 studies including a total of 7,318 patients and 85 carriers of 2846T.
GAS 1.5 (*1/c.1236G>A or *1/c.2846A>T):
Risk of grade 3 or higher fluoropyrimidine induced toxicity was 0.450381679 (See Figure 2 and Figure 4, (sum of events c.1236 + sum of events c.2846)/(sum of patients c.1236 + sum of patients c.2846) =(53+65)/(177+85)= 0.450381679) among untested-actionable DPYD GAS 1.5. Risk of death as a result of grade 3 fluoropyrimidine induced toxicity is approximately 0.0075 (75). Therefore, risk of death as a result of leukopenia is 0.450381679 x 0.0075 = 0.003377863 for untested-actionable DPYD GAS 1.5. These are given a certainty score of 3.
GAS 1.0 (*1/*2A or *1/*13):
Risk of grade 3 or higher fluoropyrimidine induced toxicity was 0.690140845 (See Figure 2 and Figure 4, sum of events *2A + sum of events *13)/(sum of patients *2A + sum of patients *13 = (43+6)/(60+11)) among untested-actionable DPYD GAS 1.0. Risk of death as a result of grade 3 fluoropyrimidine induced toxicity is approximately 0.0075 (75). Therefore, risk of death as a result of leukopenia is 0.690140845 x 0.0075 = 0.005176056 for untested-actionable DPYD GAS1.0. These are given a certainty score of 3.
GAS 0.5 (e.g. c.1236G>A/c.2846A>T or combinations of c.2846A>T or c.1236G>A with *2A or *13, example given *2A/c.2846A>T):
Risk of grade 3 or higher fluoropyrimidine induced toxicity was unable to be extracted for untested-actionable DPYD GAS 0.5 from Meulendijks D et al.(44). No suitable publication was identity in steps 3 or 4. Therefore we will assume the risk of grade 3 or higher fluoropyrimidine induced toxicity to increase linearly with decreasing GAS. Delta risk of death between GAS 1.5 and GAS 1.0 was 0.005176056 -0.003377863 = 0.0018. Therefore we estimate the risk of grade 3 or higher fluoropyrimidine induced toxicity for GAS 0.5 to be 0.005176056 – 0.0018 = 0.0034. Therefore we

estimate the risk of grade 3 or higher fluoropyrimidine induced toxicity for GAS 0.5 to be [risk of death GAS 1.5 + delta risk] = 0.005176056 + 0.0018 = 0.0070. These are given a certainty score of 0
GAS 0 (*2A/*2A or *13/*13 or *2A/*13):
Risk of grade 3 or higher fluoropyrimidine induced toxicity was unable to be extracted for untested-actionable DPYD GAS 0. from Meulendijks D et al.(44). No suitable publication was identified in steps 3 or 4. Therefore we will assume the risk of grade 3 or higher fluoropyrimidine induced toxicity to increase linearly with decreasing GAS. Delta risk of death between GAS 1.5 and GAS 1.0 was 0.005176056 -0.003377863 = 0.0018. Therefore we estimate the risk of grade 3 or higher fluoropyrimidine induced toxicity for GAS 0.5 to be [risk of death GAS 0.5 + delta risk] = 0.0070 + 0.0018 = 0.0088. These are given a certainty score of 0.

1.3.4 Absolute risk extraction tested actionables (GAS 0-1.5):

Kleinjan JP et al.(48), Henricks LM et al. (49),Lunenburg CATC et al. (50), Henricks LM et al. (51), and Deenen M et al. (55) were selected for extraction of tested-actionable groups. Only patients who receive pre-therapeutic DPYD guided fluoropyrimidine therapy were considered for risk extraction.
Kleinjan JP et al.(48) is an observational study where capecitabine was dosed based on DPYD genotype in heterozygote DPYD variant carriers. Capecitabine doses were reduced in case of a DPYD variant (DPYD*2A, c.2846A>T, DPYD*13, or c.1236G>A) and subsequently adjusted on the basis of tolerance. Results were compared with a cohort of capecitabine-treated DPYD wild-type patients. Of 185 patients eligible for analysis, 11 patients were heterozygous for a DPYD variant. A median dose escalation of 8.5% was achieved using the prespecified protocol. One DPYD variant carrier experienced a grade 3 toxicity after a dose escalation. Overall, DPYD variant carriers did not experience more, or more severe toxicities than DPYD wild-type patients. The total prevalence of severe toxicities in the wild-type group was 43.1% and is comparable with the literature.
Henricks LM et al.(49) investigated the effectiveness and safety of DPYD*2A genotype-guided dosing. A cohort of 40 prospectively identified heterozygous DPYD*2A carriers, treated with a ~50% reduced fluoropyrimidine dose, was identified. The frequency of severe (grade ≥ 3) treatment-related toxicity was compared to 1] a cohort of 1606 wild-type patients treated with full dose and 2] a cohort of historical controls derived from literature, i.e. 86 DPYD*2A variant carriers who received a full fluoropyrimidine dose. For 37 out of 40 DPYD*2A carriers, a matched control could be identified. Compared to matched controls, risk of severe fluoropyrimidine-related toxicity in DPYD*2A carriers treated with reduced dose was 18%, comparable to wild-type patients (23%, p = 0.57) and significantly lower than the risk of 77% in DPYD*2A carriers treated with full dose (p < 0.001).40 patients with genotype *1/*2A and treated with an approximately 50% reduced fluoropyrimidine dose were compared to patients without *2A and to *1/*2A treated with full dose. To compare safety, *1/*2A patients treated with a reduced dose were compared with 1606 patients without *2A treated with full dose from Deenen 2016 and with 86 historical controls (*2A-carriers treated with full dose; including the historical controls in Deenen 2016).
Lunenburg CATC et al. (50) investigated the risk of severe toxicity in DPYD variant allele carriers receiving chemoradiation. Medical records of 828 patients who received fluoropyrimidine based chemoradiation (FP-based CRT) were reviewed from three centres. Severe (grade ≥III) toxicity in DPYD variant allele carriers receiving upfront dose reductions according to pharmacogenetic dosing guidelines and DPYD variant allele carriers not receiving dose reductions was compared with DPYD wild-type patients receiving standard dose. DPYD variant allele carriers treated with standard dosages (N = 34) showed an increased risk of severe gastrointestinal (adjusted OR = 2.58, confidence interval [CI] = 1.02-6.53, P = 0.045) or severe haematological (adjusted OR = 4.19, CI = 1.32-13.25,

P = 0.015) toxicity compared with wild-type patients (N = 771). DPYD variant allele carriers who received dose reductions (N = 22) showed a comparable frequency of severe gastrointestinal toxicity compared with wild-type patients, but more (not statistically significant) severe haematological toxicity. Hospitalisations for all DPYD variant allele carriers were comparable, independent of dose adjustments; however, the mean duration of hospitalisation was significantly shorter in the dose reduction group (P = 0.010).
Henricks LM et al.(51) is a prospective, multicentre, safety analysis in 17 hospitals in the Netherlands, the study population consisted of adult patients (≥18 years) with cancer who were intended to start on a fluoropyrimidine-based anticancer therapy (capecitabine or fluorouracil as single agent or in combination with other chemotherapeutic agents or radiotherapy). Patients with all tumour types for which fluoropyrimidine-based therapy was considered in their best interest were eligible. We did prospective genotyping for DPYD*2A, c.2846A>T, c.1679T>G, and c.1236G>A. Heterozygous DPYD variant allele carriers received an initial dose reduction of 25% (c.2846A>T and c.1236G>A) or 50% (DPYD*2A and c.1679T>G), and DPYD wild-type patients were treated according to the current standard of care. The primary endpoint of the study was the frequency of severe (National Cancer Institute Common Terminology Criteria for Adverse Events version 4.03 grade ≥3) overall fluoropyrimidine-related toxicity across the entire treatment duration. Toxicity incidence was compared between DPYD variant allele carriers and DPYD wild-type and relative risks (RRs) for severe toxicity were compared between the current study and a historical cohort of DPYD variant allele carriers treated with full dose fluoropyrimidine-based therapy (derived from a previously published meta-analysis). Of 1103 evaluable patients, 85 (8%) were heterozygous DPYD variant allele carriers, and 1018 (92%) were DPYD wild-type patients. Overall, fluoropyrimidine-related severe toxicity was higher in DPYD variant carriers (33 [39%] of 85 patients) than in wild-type patients (231 [23%] of 1018 patients; p=0·0013). The RR for severe fluoropyrimidine-related toxicity was 1·31 (95% CI 0·63-2·73) for genotype-guided dosing compared with 2·87 (2·14-3·86) in the historical cohort for DPYD*2A carriers, no toxicity compared with 4·30 (2·10-8·80) in c.1679T>G carriers, 2·00 (1·19-3·34) compared with 3·11 (2·25-4·28) for c.2846A>T carriers, and 1·69 (1·18-2·42) compared with 1·72 (1·22-2·42) for c.1236G>A carriers.
Deenen M et al.(55) determines the feasibility, safety, and cost of DPYD*2A genotype-guided dosing. Patients intended to be treated with fluoropyrimidine-based chemotherapy were prospectively genotyped for DPYD*2A before start of therapy. Variant allele carriers received an initial dose reduction of ≥ 50% followed by dose titration based on tolerance. Toxicity was the primary end point and was compared with historical controls (ie, DPYD*2A variant allele carriers receiving standard dose described in literature) and with DPYD*2A wild-type patients treated with the standard dose in this study.
A total of 2,038 patients were prospectively screened for DPYD*2A, of whom 22 (1.1%) were heterozygous polymorphic. DPYD*2A variant allele carriers were treated with a median dose-intensity of 48% (range, 17% to 91%). The risk of grade ≥ 3 toxicity was thereby significantly reduced from 73% (95% CI, 58% to 85%) in historical controls (n = 48) to 28% (95% CI, 10% to 53%) by genotype-guided dosing (P < .001); drug-induced death was reduced from 10% to 0%. Adequate treatment of genotype-guided dosing was further demonstrated by a similar incidence of grade ≥ 3 toxicity compared with wild-type patients receiving the standard dose (23%; P = .64) and by similar systemic fluorouracil (active drug) exposure.

GAS 1.5 (*1/c.1236G>A or *1/c.2846A>T):

	Kleinjan JP et al.(48)*	Henricks LM et al.(49)	Lunenburg CATC et al.(50)	Henricks LM et al.(51)	Deenen M et al.(55)	Total

Number of patients GAS 1.5	11	Not reported	12	51+17=68	Not reported	91
Number of events	3	Not reported	5	3+1=4	Not reported	12
Overall absolute risk	-	-	-	-	-	0.131868

*Four (36.4%) were DPYD*2A heterozygous, one (9.1%) was c.2846A >T heterozygous, and the remaining six (54.5%) were c.1236G > A heterozygous. No DPYD*13 variant carriers were identified.

Risk of death as a result of grade 3 fluoropyrimidine induced toxicity is approximately 0.0075 (75). Therefore, risk of death as a result of leukopenia is 0.131868 x 0.0075 = 0.0010 for untested-actionable DPYD GAS 1.5. These are given a certainty score of 1.

GAS 1.0 (*1/*2A or *1/*13):

	Kleinjan JP et al.(48)*	Henricks LM et al.(49)	Lunenburg CATC et al. (50)	Henricks LM et al.(51)**	Deenen M et al.(55)	Total
Number of patients GAS 1.0	11	40	11	16+1=17	18	97
Number of events	3	7	5	0	2	17
Overall absolute risk	-	-	-	-	-	0.175258

*Four (36.4%) were DPYD*2A heterozygous, one (9.1%) was c.2846A >T heterozygous, and the remaining six (54.5%) were c.1236G > A heterozygous. No DPYD*13 variant carriers were identified.

**Only limit to prospectively genotyped patient for *2A, exclude historical controls from Deenen et al.

Risk of death as a result of grade 3 fluoropyrimidine induced toxicity is approximately 0,0075 (75). Therefore, risk of death as a result of leukopenia is 0.175258 x 0.0075 = 0.0013 for untested-actionable DPYD GAS 1.0. These are given a certainty score of 1.

GAS 0.5 (e.g. c.1236G>AA/c.2846A>T or combinations of c.2846A>T or c.1236G>A with *2A or *13, example given *2A/c.2846A>T):

	Kleinjan JP et al.(48)	Henricks LM et al.(49)	Lunenburg CATC et al. (50)	Henricks LM et al.(51)	Deenen M et al.(55)	Total
Number of patients GAS 0.5	Not reported	Not reported	Not reported	Not reported	Not reported	Not applicable
Number of events	Not reported	Not reported	Not reported	Not reported	Not reported	Not applicable
Overall absolute risk	-	-	-	-	-	Not applicable

Risk of grade 3 or higher fluoropyrimidine induced toxicity was unable to be extracted for untested-actionable DPYD GAS 0.5 from (48) (49, 50) (51, 55). No suitable publication was identified in step 5. Therefore we will assume the risk of grade 3 or higher fluoropyrimidine induced toxicity is equal to the mean risk of death of GAS 1.5 and 1. The mean of these is 0.0012. These are given a certainty score of 0.

GAS 0.0 (*2A/*2A or *13/*13 or *2A/*13):

	Kleinjan JP et al.(48)	Henricks LM et al.(49)	Lunenburg CATC et al. (50)	Henricks LM et al.(51)	Deenen M et al.(55)	Total
Number of patients GAS 0	Not reported	Not reported	Not reported	Not reported	Not reported	Not applicable
Number of events	Not reported	Not reported	Not reported	Not reported	Not reported	Not applicable
Overall absolute risk	-	-	-	-	-	Not applicable

Risk of grade 3 or higher fluoropyrimidine induced toxicity was unable to be extracted for untested-actionable DPYD GAS 0.5 from (48) (49, 50) (51, 55). No suitable publication was identified in step 5. Therefore we will assume the risk of grade 3 or higher fluoropyrimidine induced toxicity is equal to the mean risk of death of GAS 1.5 and 1. The mean of these is 0.0012. These are given a certainty score of 0.

1.3.5 Conclusion of selected publications and absolute risks extracted:

		Actionability	Untested	Ref	CS	Tested	Ref	CS
Capecitabine/5-FU	DPYD GAS 0	yes	0.0088	-	0	0.0012	-	0
Capecitabine/5-FU	DPYD GAS 0.5	yes	0.0070	-	0	0.0012	-	0
Capecitabine/5-FU	DPYD GAS 1.0	yes	0.005176056	(44)	3	0.0013	(48) (49, 50) (51, 55)	1
Capecitabine/5-FU	DPYD GAS 1.5	yes	0.003377863	(44)	3	0.0010	(48) (50) (51)	1
Capecitabine/5-FU	DPYD GAS 2	no	0.002430066	(44)	3	0.002430066	(44)	3

Ref: Reference; CS: Certainty score

1.4 *CYP2C19*-CLOPIDOGREL

1.4.1 Publication selection

Risk analysis: https://kennisbank.knmp.nl/files/farmacogenetica/2548-2549-2550.PDF

There is a DPWG guideline for the combined indications of percutaneous coronary intervention (PCI), stoke and transient ischemic attack (TIA). Therefore, we have chosen to select publications and perform subsequent risk extraction for all three indications combined.

	Steps performed systematically to select suitable publication(s) form which extraction is performed	Publication(s) selection	
1	Publications reporting predicted phenotype group: quality score 4, powered on mortality	**Study**	**Conclusion**
		Niu X et al. (76)	Not powered on mortality. Not selected.
		Jang JS et al. (77)	Not powered on mortality. Not selected.
		Pan Y et al. (78)	Not powered on mortality. Not selected.
		Sorich MJ et al. (79)	Not powered on mortality. Not selected.
		Mao L et al. (80)	Not powered on mortality. Not selected.
		Li Y et al. (81)	Not powered on mortality. Not selected.
		Holmes MV et al. (82)	Not powered on mortality. Not selected.
		Liu YP et al. (83)	Not powered on mortality. Not selected.
		Mega JL et al.(84)	Not powered on mortality. Not selected.
		Simon T et al. (85)	Not powered on mortality. Not selected.
		Collet JP et al. (86)	Not powered on mortality. Not selected.
		Simon T et al. (87)	Not powered on mortality. Not selected.
		Shen DL et al. (88)	Not powered on mortality. Not selected.
		Mega JL et al.(89)	Not powered on mortality. Not selected.

<table>
<tr><td></td><td></td><td>
<table>
<tr><td>Geisler T et al.(90)</td><td>Not powered on mortality. Not selected.</td></tr>
<tr><td>Chen BL et al.(91)</td><td>Not powered on mortality. Not selected.</td></tr>
<tr><td>Kim KA et al. (92)</td><td>Not powered on mortality. Not selected.</td></tr>
<tr><td>Malek LA et al.(93)</td><td>Not powered on mortality. Not selected.</td></tr>
<tr><td>Trenk D et al. (94)</td><td>Not powered on mortality. Not selected.</td></tr>
<tr><td>Fontana P et al.(95)</td><td>Not powered on mortality. Not selected.</td></tr>
<tr><td>Hulot JS et al.(96)</td><td>Not powered on mortality. Not selected.</td></tr>
</table>
Conclusion:
No publication was selected therefore we will continue to the next step.
</td></tr>
<tr><td>2</td><td>Publications reporting predicted phenotype group: quality score 4</td><td>
<table>
<tr><th>Study</th><th>Conclusion</th></tr>
<tr><td>Niu X et al. (76)</td><td>Usable risk data: Yes

Representative: No, predominantly studies performed in Asia

Not selected.</td></tr>
<tr><td>Jang JS et al. (77)</td><td>Usable risk data: Yes (genetic variant in most studies is *2

Representative: Yes

Not selected. Another meta-analysis is more recent (2012).</td></tr>
<tr><td>Pan Y et al. (78)</td><td>Usable risk data: Yes (*2, 3, 17, 1)

Representative: No

Not selected.</td></tr>
<tr><td>Sorich MJ et al. (79)</td><td>Usable risk data: Yes (*2, 3, 17, 1)

Representative: Yes

Most recent meta-analysis (2014). Selected for extraction of untested groups.</td></tr>
<tr><td>Mao L et al. (80)</td><td>Usable risk data: Yes (loss of function *2-*8)

Representative: Yes</td></tr>
</table>
</td></tr>
</table>

	Another meta-analysis is more recent (2013).
Li Y et al. (81)	Usable risk data: No (only *17)<br><br>Representative: Yes<br><br>Not selected.
Holmes MV et al. (82)	Usable risk data: Yes (any loss of function allele)<br><br>Representative: Yes<br><br>Not selected. Another meta-analysis is more recent (2011).
Liu YP et al. (83)	Usable risk data: Yes (Any loss of function allele)<br><br>Representative: Yes<br><br>Not selected. Another meta-analysis is more recent (2011).
Mega JL et al.(84)	Usable risk data: Yes (only *2 loss of function)<br><br>Representative: Yes<br>Not selected. Another meta-analysis is more recent.
Simon T et al. (85)	Usable risk data: No<br><br>Representative: No (healthy subjects)<br><br>Not selected.
Collet JP et al. (86)	Usable risk data: Yes (only *2 loss of function)<br><br>Representative: No, young patients (<45 years)<br><br>Not selected.
Simon T et al. (87)	Usable risk data: Yes (multiple loss of function alleles)<br><br>Representative: Yes<br><br>Not selected. Included in meta-analysis by Sorich MJ et al.
Shen DL et al. (88)	Representative: No, Chinese population.<br><br>Not selected.
Mega JL et al.(89)	Usable risk data: Yes

<table>
<tr><td></td><td></td><td>
<table>
<tr><td></td><td>Representative: Yes

Not selected. Included in meta-analysis by Sorich MJ et al.</td></tr>
<tr><td>Geisler T et al.(90)</td><td>Usable risk data: No

Not selected.</td></tr>
<tr><td>Chen BL et al.(91)</td><td>Representative: No, healthy volunteers

Not selected.</td></tr>
<tr><td>Kim KA et al. (92)</td><td>Representative: No, healthy volunteers

Not selected.</td></tr>
<tr><td>Malek LA et al.(93)</td><td>Representative: Yes

Usable risk data: No, reports on CADP-CT</td></tr>
<tr><td>Trenk D et al. (94)</td><td>Representative: Yes

Usable risk data: No, reports on residual platelet aggregation</td></tr>
<tr><td>Fontana P et al.(95)</td><td>Representative: No, healthy volunteers

Not selected.</td></tr>
<tr><td>Hulot JS et al.(96)</td><td>Representative: No, healthy volunteers.

Not selected.</td></tr>
</table>
Conclusion:
Only the risks for the untested groups can be obtained with this step. We have selected the most recent suitable meta-analysis by Sorich MJ et al. (79) for extraction of data for untested groups.
</td></tr>
<tr><td>3</td><td>Publications reporting predicted phenotype group: quality score 3, powered on mortality</td><td>
<table>
<tr><td>Study</td><td>Conclusion</td></tr>
<tr><td>Lee CR et al.(97)</td><td>Not powered on mortality. Not selected.</td></tr>
<tr><td>Zhong Z et al. (98)</td><td>Not powered on mortality. Not selected.</td></tr>
<tr><td>Wu Y et al. (99)</td><td>Not powered on mortality. Not selected.</td></tr>
<tr><td>Cavallari LH et al. (100)</td><td>Not powered on mortality. Not selected.</td></tr>
</table>
</td></tr>
</table>

			Lin Y et al. (101)	Not powered on mortality. Not selected.	
			Deiman BA et al. (102)	Not powered on mortality. Not selected.	
			Wang Y et al. (103)	Not powered on mortality. Not selected.	
			Ogawa H et al. (104)	Not powered on mortality. Not selected.	
			Xiong R et al. A (105)	Not powered on mortality. Not selected.	
			Xie X et al.(106)	Not powered on mortality. Not selected.	
			Collet JP et al. (107)	Not powered on mortality. Not selected.	
			Bonello-Palot N et al. (108)	Not powered on mortality. Not selected.	
			Shuldiner AR et al. (109)	Not powered on mortality. Not selected.	
			Frére C et al. (110)	Not powered on mortality. Not selected.	
			Aleil B et al.(111)	Not powered on mortality. Not selected.	
			Sibbing D et al. (112)	Not powered on mortality. Not selected.	
			Brackbill ML et al. (113)	Not powered on mortality. Not selected.	
			Giusti B et al. (114)	Not powered on mortality. Not selected.	
			Umemura K et al. (115)	Not powered on mortality. Not selected.	
			Frére C et al. (116)	Not powered on mortality. Not selected.	
			Fontana P et al. (117)	Not powered on mortality. Not selected.	
			Giusti B et al. (118)	Not powered on mortality. Not selected.	
			Brandt JT et al. (119)	Not powered on mortality. Not selected.	

<table>
<tr><td></td><td></td><td>Conclusion:

No publication was selected therefore we will continue to the next step.</td></tr>
<tr><td>4</td><td>Publications reporting predicted phenotype group: quality score 3</td><td>
<table>
<tr><th>Study</th><th>Conclusion</th></tr>
<tr><td>Lee CR et al.(97)</td><td>Usable risk data: Yes (MACE)
Representative: Yes
Selected for extraction of tested groups.</td></tr>
<tr><td>Zhong Z et al. (98)</td><td>Representative: No, Chinese patients.

Not selected.</td></tr>
<tr><td>Wu Y et al. (99)</td><td>Representative: No, Chinese patients.
Not selected.</td></tr>
<tr><td>Cavallari LH et al. (100)</td><td>Usable risk data: Yes
Representative: Yes

Selected for extraction of tested groups.</td></tr>
<tr><td>Lin Y et al. (101)</td><td>Representative: No, Chinese patients.

Not selected.</td></tr>
<tr><td>Deiman BA et al. (102)</td><td>Usable risk data: No, only PM selected.
Representative: Yes

Not selected.</td></tr>
<tr><td>Wang Y et al. (103)</td><td>Representative: No, Chinese patients.

Not selected.</td></tr>
<tr><td>Ogawa H et al. (104)</td><td>Representative: No, Japanese patients.

Not selected.</td></tr>
<tr><td>Xiong R et al. A (105)</td><td>Representative: No, Chinese patients.

Not selected.</td></tr>
<tr><td>Xie X et al.(106)</td><td>Representative: No, Chinese patients.

Not selected.</td></tr>
</table>
</td></tr>
</table>

		Collet JP et al. (107)	Representative: No, only young and male patients selected.<br>Usable risk data: No<br><br>Not selected.
		Bonello-Palot N et al. (108)	Usable risk data: No. Not genotype-guided.<br><br>Representative: Yes.<br><br>Not selected.
		Shuldiner AR et al. (109)	Usable risk data: No. Not genotype-guided.<br>Not selected.
		Frére C et al. (110)	Usable risk data: No. Not genotype-guided.<br>Not selected.
		Aleil B et al.(111)	Usable risk data: No. Not genotype-guided.<br>Not selected.
		Sibbing D et al. (112)	Usable risk data: No. Not genotype-guided.<br>Not selected.
		Brackbill ML et al. (113)	Usable risk data: No. Not genotype-guided.<br>Not selected.
		Giusti B et al. (114)	Usable risk data: No. Not genotype-guided.<br>Not selected.
		Umemura K et al. (115)	Usable risk data: No. Not genotype-guided.<br>Not selected.
		Frére C et al. (116)	Usable risk data: No. Not genotype-guided.<br>Not selected.
		Fontana P et al. (117)	Usable risk data: No. Not genotype-guided.<br>Not selected.
		Giusti B et al. (118)	Usable risk data: No. Not genotype-guided.<br>Not selected.
		Brandt JT et al. (119)	Usable risk data: No. Not genotype-guided.<br>Not selected.
		Conclusion:<br>We have selected 2 publications (Lee CR et al.(97)and Cavallari LH et al. (100)) for the extraction of data for tested groups.	

5	Perform literature review in review of a usable study regarding the relevant DGI	Not applicable

1.4.2 Absolute risk extraction non-actionables (UM and EM):

Sorich MJ et al.(79) was selected for extraction of non-actionables groups.
Sorich MJ et al. (79) is a meta-analysis assessing the association between CYP2C19 LoF allele carriage and major cardiovascular outcomes differs based on the ethnic population and the clopidogrel indication. Of the 23 studies in this meta-analysis, 15 studies were also included in the Mao 2014 meta-analysis, 9 in the Jang 2012 meta-analysis, 13 in the Holmes 2011 meta-analysis and 10 in the Liu 2011 meta-analysis. Five of the articles in the meta-analysis were also included separately in this risk analysis (Trenk 2008, Giusti 2009, Mega 2009, Sibbing 2009 and Simon 2009).
Meta-analysis of 24 studies (23 publications) including a total of 36,076 patients using clopidogrel. 16 studies were performed in Caucasian populations (ntotal = 26,059), 8 in Asian populations (ntotal = 10,017). The meta-analysis only incorporated studies including n ≥ 500 patients.
Major adverse cardiovascular outcomes (death, cardiovascular death, nonfatal myocardial infarction, and nonfatal stroke): Sorich MH et al. (79) has combined the *CYP2C19* UM and EMs in one group, therefore we will also perform risk extraction for UM and EMs combined. Risk of major adverse cardiovascular outcomes was 0.091849866 (See Figure 2, white non-PCI + white PCI, sum of events/sum of patients = 449+1264/5152+13498) among non-actionable CYP2C19 UM and EMs. The risk of cardiovascular death in MACE is 0.34. Therefore, risk of death as a result of adverse cardiovascular events is 0.091849866 x 0.34 = 0.03146708 for non-actionable CYP2C19 UM and EMs. These are given a certainty score of 3.

1.4.3 Absolute risk extraction untested actionables (IM and PM):

Sorich MJ et al. (79) was selected for extraction of untested actionable groups.
Sorich MJ et al. (79) is a meta-analysis assessing the association between CYP2C19 LoF allele carriage and major cardiovascular outcomes differs based on the ethnic population and the clopidogrel indication. Of the 23 studies in this meta-analysis, 15 studies were also included in the Mao 2014 meta-analysis, 9 in the Jang 2012 meta-analysis, 13 in the Holmes 2011 meta-analysis and 10 in the Liu 2011 meta-analysis. Five of the articles in the meta-analysis were also included separately in this risk analysis (Trenk 2008, Giusti 2009, Mega 2009, Sibbing 2009 and Simon 2009).
Meta-analysis of 24 studies (23 publications) including a total of 36,076 patients using clopidogrel. 16 studies were performed in Caucasian populations (n total = 26,059), 8 in Asian populations (n total = 10,017). The meta-analysis only incorporated studies including n ≥ 500 patients.
Major adverse cardiovascular outcomes (death, cardiovascular death, nonfatal myocardial infarction, and nonfatal stroke): Sorich MH et al. (79) has combined the CYP2C19 IMs and PMs in one group, therefore we will also perform risk extraction for IM and PMs combined. Risk of major adverse cardiovascular outcomes was 0.107436901 (See Figure 2, white non-PCI + white PCI, sum of events/sum of patients = 177+619/1891+5518) among untested actionable CYP2C19 IMs and PMs. The risk of cardiovascular death in MACE is 0.34 (120). Therefore, risk of death as a result of adverse cardiovascular events is 0.107436901 x 0.34 = 0.036807086 for untested actionable CYP2C19 IMs and PMs. These are given a certainty score of 3.

1.4.4 Absolute risk extraction tested actionables (IM and PM):

Lee CR et al.(97) and Cavallari LH et al. (100) were selected for the extraction of data for tested groups. Both studies have given CYP2C19 IMs and PMs alternative therapies with ticagrelor and prasugrel.

Lee CR et al.(97) assessed the feasibility, sustainability and clinical impact of using CYP2C19 genotype-guided dual antiplatelet therapy (DAPT) selection in practice remains unclear. This single-center observational study was conducted in 1,193 patients who underwent PCI and received DAPT following implementation of an algorithm that recommends CYP2C19 testing in high-risk patients and alternative DAPT (prasugrel or ticagrelor) in LOF allele carriers. The frequency of genotype testing and alternative DAPT selection were the primary implementation endpoints. Risk of major adverse cardiovascular or cerebrovascular (MACCE) and clinically significant bleeding events over 12 months were compared across genotype and DAPT groups. CYP2C19 genotype was obtained in 868 (72.8%) patients. Alternative DAPT was prescribed in 186 (70.7%) LOF allele carriers.
Cavallari LH et al. (100) is a multicenter pragmatic investigation assessed outcomes following clinical implementation of
CYP2C19 genotype–guided antiplatelet therapy after percutaneous coronary intervention (PCI). After clinical genotyping, each institution recommended alternative antiplatelet therapy (prasugrel, ticagrelor) in PCI patients with a loss-of-function allele. Major adverse cardiovascular events (defined as myocardial infarction, stroke, or death) within 12 months of PCI were compared between patients with a loss-of-function allele prescribed clopidogrel versus alternative therapy. Risk was also compared between patients without a loss-of-function allele and loss-of-function allele carriers prescribed alternative therapy. Among 1,815 patients, 572 (31.5%) had a loss-of-function allele. The risk for major adverse cardiovascular events was significantly higher in patients with a loss-of-function allele prescribed clopidogrel versus alternative therapy(23.4 vs. 8.7 per 100 patient-years; adjusted hazard ratio: 2.26; 95% confidence interval: 1.18 to 4.32; p = 0.013). Similar results were observed among 1,210 patients with acute coronary syndromes at the time of PCI (adjusted hazard ratio:2.87; 95% confidence interval: 1.35 to 6.09; p = 0.013).
Lee CR et al.(97) and Cavallari LH et al. have combined the CYP2C19 IM and PMs in one group, therefore we will also perform risk extraction for IM and PMs combined.
Lee CR et al.(97):
Risk of major cardiovascular events was 0.053763441 (See Figure 3A, LOF-alt, n events/n patients (extracted from Fig 1A) =10/186) among actionable CYP2C19 IM and PMs.
Cavallari LH et al. (100):
Risk of major cardiovascular events was 0.080924855 (See Table 3, LOF-alternative, n events/n patients =28/346) among actionable CYP2C19 IM and PMs.

	Lee CR et al.(97)	Cavallari LH et al. (100)	Total
Number of MACE	10	28	38
Number of patients CYP2C19 IM or PM who received alternative P2Y12 inhibitor	186	346	532
Overall absolute risk	-	-	0.071428571

The risk of cardiovascular death in MACE is 0.34 (120). Therefore, risk of death as a result of adverse cardiovascular events is 0.071428571x 0.34 = 0.024470899 for tested actionable CYP2C19 IMs and PMs. These are given a certainty score of 1.

1.4.5 Conclusion of selected publications and absolute risks extracted:

		Actionability	Untested	Ref	CS	Tested	Ref	CS
Clopidogrel	CYP2C19 EM	No	0.031467084	(79)	3	0.031467084	(79)	3

Clopidogrel	CYP2C19 IM	Yes	0.036807086	(79)	3	0.024470899	(97, 100)	1
Clopidogrel	CYP2C19 PM	Yes	0.036807086	(79)	3	0.024470899	(97, 100)	1
Clopidogrel	CYP2C19 UM	no	0.031467084	(79)	3	0.031467084	(79)	3

Ref: Reference; CS: certainty score

1.5 *UGT1A1*-IRINOTECAN

1.5.1 Publication selection

Risk analysis: https://kennisbank.knmp.nl/files/farmacogenetica/1691-1692.PDF

	Steps performed systematically to select suitable publication(s) form which extraction is performed	Publication(s) selection
1	Publications reporting predicted phenotype group: quality score 4, powered on mortality	**Study** / **Conclusion**<br>Chen X et al. (121): Not powered on mortality. Not selected.<br>Liu XH et al. (122): Not powered on mortality. Not selected.<br>Han FF et al. (123): Not powered on mortality. Not selected.<br>Chen YJ et al. (124): Not powered on mortality. Not selected.<br>Liu X et al.(125): Not powered on mortality. Not selected.<br>Hu ZY et al. (126): Not powered on mortality. Not selected.<br>Hu ZY et al. (127): Not powered on mortality. Not selected.<br>Hoskins JM et al. (128): Not powered on mortality. Not selected.<br>Dias MM et al. (129): Not powered on mortality. Not selected.<br>Liu X et al. (130): Not powered on mortality. Not selected.<br>Dias MM et al.(131): Not powered on mortality. Not selected.<br>Denlinger CS et al.(132): Not powered on mortality. Not selected.<br>Conclusion:<br>No publication was selected therefore we will continue to the next step.
2	Publications reporting predicted phenotype group: quality score 4	**Study** / **Conclusion**<br>Chen X et al. (121): Usable risk data: Yes<br>Representative: No, Asian patients.<br>Not selected.

		Liu XH et al. (122)	Usable risk data: Yes<br>Representative: No, less than 50% of the studies performed in Caucasian population.<br><br>Not selected.
		Han FF et al. (123)	Usable risk data: No (only ORs)<br>Representative: No, Asian patients.<br>Not selected.
		Chen YJ et al. (124)	Usable risk data: No<br>Representative: No, Asian patients.<br>Not selected.
		Liu X et al.(125)	Usable risk data: Yes<br>Representative: Yes<br><br>**Selected for extraction of untested groups.**
		Hu ZY et al. (126)	Usable risk data: No (only ORs)<br>Representative: Yes<br>Not selected.
		Hu ZY et al. (127)	Usable risk data: Yes<br>Representative: Yes<br><br>Not selected. Another meta-analysis is more recent.
		Hoskins JM et al. (128)	Usable risk data: No, only looks at *28/*28 vs. *1/*1 + *28/*1<br>Representative: Yes<br><br>Not selected.
		Dias MM et al. (129)	Usable risk data: No<br><br>Not selected.
		Liu X et al. (130)	Usable risk data: No<br><br>Representative: Yes<br>Not selected.
		Dias MM et al.(131)	Usable risk data: No<br><br>Not selected.
		Denlinger CS et al.(132)	Usable risk data: No<br>Not selected.
		Conclusion:	

		Liu X et al.(125) was selected for extraction of untested and non-actionable groups. Therefore we will continue with the next step to obtain the data for the tested groups.
3	Publications reporting predicted phenotype group: quality score 3, powered on mortality	(nested table below)

**Study**	**Conclusion**
Lu CY et al. (133)	Not powered on mortality. Not selected.
Goetz MP et al. (134)	Not powered on mortality. Not selected.
Kweekel DM et al. (135)	Not powered on mortality. Not selected.
Liu CY et al. (136)	Not powered on mortality. Not selected.
Lankisch TO et al. (137)	Not powered on mortality. Not selected.
Minami H et al. (138)	Not powered on mortality. Not selected.
Stewart CF et al. (139)	Not powered on mortality. Not selected.
Côté JF et al. (140)	Not powered on mortality. Not selected.
Ramchandani RP et al..(141)	Not powered on mortality. Not selected.
Zárate Romero R et al. (142)	Not powered on mortality. Not selected.
de Jong FA et al. (143)	Not powered on mortality. Not selected.
Toffoli G et al. (144)	Not powered on mortality. Not selected.
Han JY et al.(145)	Not powered on mortality. Not selected.
McLeod HL et al. (146)	Not powered on mortality. Not selected.
Massacesi C et al. (147)	Not powered on mortality. Not selected.
Wright MA et al. (148)	Not powered on mortality. Not selected.
Kweekel DM etal.	Not powered on mortality. Not selected.
Soepenberg O et al. (149)	Not powered on mortality. Not selected.

Zhou Q et al. (150)	Not powered on mortality. Not selected.
Carlini LE et al. (151)	Not powered on mortality. Not selected.
Kitagawa C et al. (152)	Not powered on mortality. Not selected.
Marcuello E et al. (153)	Not powered on mortality. Not selected.
Rouits E et al.(154)	Not powered on mortality. Not selected.
Paoluzzi L et al. (155)	Not powered on mortality. Not selected.
Sai K et al. (156)	Not powered on mortality. Not selected.
Innocenti F et al. (157)	Not powered on mortality. Not selected.
Font A et al. (158)	Not powered on mortality. Not selected.
Mathijssen RH et al. (159)	Not powered on mortality. Not selected.
Iyer L et al. (160)	Not powered on mortality. Not selected.
Ando Y et al. (161)	Not powered on mortality. Not selected.

Conclusion:
No publication was selected therefore we will continue to the next step.

4 Publications reporting predicted phenotype group: quality score 3

**Study**	**Conclusion**
Lu CY et al. (133)	Representative: No, Taiwanese patients.<br>Not selected.
Goetz MP et al. (134)	Representative: Yes<br>Usable risk data: No.<br>Not selected.
Kweekel DM et al. (135)	Representative: Yes<br>Usable risk data: No, not genotype-guided.<br>Not selected.
Liu CY et al. (136)	Representative: No, Chinese patients.<br>Not selected.
Lankisch TO et al. (137)	Representative: Yes

	Usable risk data: No, not genotype-guided.<br>Not selected.
Minami H et al. (138)	Representative: No, Japanese patients.<br>Not selected.
Stewart CF et al. (139)	Representative: No, pediatric population.<br>Usable risk data: No, not genotype-guided<br>Not selected.
Côté JF et al. (140)	Usable risk data: No, not genotype-guided<br>Not selected.
Ramchandani RP et al..(141)	Usable risk data: No, not genotype-guided<br>Not selected.
Zárate Romero R et al. (142)	Usable risk data: No, not genotype-guided<br>Not selected.
de Jong FA et al. (143)	Usable risk data: No, not genotype-guided<br>Not selected.
Toffoli G et al. (144)	Usable risk data: No, not genotype-guided<br>Not selected.
Han JY et al.(145)	Usable risk data: No, not genotype-guided<br>Not selected.
McLeod HL et al. (146)	Usable risk data: No, not genotype-guided<br>Not selected.
Massacesi C et al. (147)	Usable risk data: No, not genotype-guided<br>Not selected.
Wright MA et al. (148)	Usable risk data: No, not genotype-guided<br>Not selected.
Kweekel DM etal.	Usable risk data: No, not genotype-guided<br>Not selected.
Soepenberg O et al. (149)	Usable risk data: No, not genotype-guided<br>Not selected.
Zhou Q et al. (150)	Usable risk data: No, not genotype-guided<br>Not selected.

Carlini LE et al. (151)	Usable risk data: No, not genotype-guided<br>Not selected.
Kitagawa C et al. (152)	Usable risk data: No, not genotype-guided<br>Not selected.
Marcuello E et al. (153)	Usable risk data: No, not genotype-guided<br>Not selected.
Rouits E et al.(154)	Usable risk data: No, not genotype-guided<br>Not selected.
Paoluzzi L et al. (155)	Usable risk data: No, not genotype-guided<br>Not selected.
Sai K et al. (156)	Usable risk data: No, not genotype-guided<br>Not selected.
Innocenti F et al. (157)	Usable risk data: No, not genotype-guided<br>Not selected.
Font A et al. (158)	Usable risk data: No, not genotype-guided<br>Not selected.
Mathijssen RH et al. (159)	Usable risk data: No, not genotype-guided<br>Not selected.
Iyer L et al. (160)	Usable risk data: No, not genotype-guided<br>Not selected.
Ando Y et al. (161)	Usable risk data: No, not genotype-guided<br>Not selected.

Conclusion:
No publication was selected therefore we will continue to the next step.

5 Perform literature review in review of a usable study regarding the relevant DGI

**Search strategy pubmed**	**Date literature search**
Irinotecan[Title] AND (UGT1A1[Title] OR Pharmacogenetic[Title] OR Pharmacogenetics [Title] OR genotype[Title] OR genotypes[Title] OR polymorphism[Title] OR polymorphisms[Title])	18-12-2019

Conclusion:

We found no intervention studies through our own literature search. Therefore, we estimated the absolute risk on death to be equal to that of non-actionables. These are given a certainty score of 0.

1.5.2 Absolute risk extraction non-actionables (*1/*1, *1/*28 and IM):

Liu X et al.(125)is a meta-analysis of 16 studies including a total of 2,328 mainly Caucasian patients with colorectal cancer. The outcome measure was grade 3-4 toxicity.

Neutropenia:
Risk of grade 3 or higher neutropenia was 0.1121 (See Figure 2, b, high IRI, sum of events/sum of patients = 72/642) among non-actionable *1/*1. Risk of drug-related death as a result of myelosuppression is 0.00949 (section treatment related deaths: 1.3% died of treatment related effects, of which 73% was associated with myelosuppression) (162). Therefore, risk of death as a result of grade 3 neutropenia is 0.1121 x .,00949 = 0.001064299 for non-actionable *1/*1. These are given a certainty score of 3.

Risk of grade 3 or higher neutropenia was 0.1865 (See Figure 3, b, high IRI, sum of events/sum of patients = 102/547) among non-actionable *1/*28 and IMs. Risk of death as a result of myelosupression is 0.00949 (section treatment related deaths: 1.3% died of treatment related effects, of which 73% was associated with myelosuppression) (162). Therefore, risk of death as a result of grade 3 neutropenia is 0.1865 x 0.00949 = 0.001769616 for non-actionable *1/*28 and IM. These are given a certainty score of 3.

Diarrhoea:

Risk of grade 3 or higher diarrhoea was 0.1109 (See Figure 4, b, high IRI, sum of events/sum of patients = 73/658) among non-actionable *1/*1. Risk of drug-related death as a result of diarrhoea is 0.001363473 (section treatment related deaths sum of patients death of diarrhoea/total patients = 19/13935) . Therefore, risk of death as a result of grade 3 diarrhoea is 0.1109 x 0.0013 = 0.000151267 for non-actionable *1/*1. These are given a certainty score of 3.

Risk of grade 3 or higher diarrhoea was 0.1473 (See Figure 5, b, high IRI, sum of events/sum of patients = 80/543) among non-actionable *1/*28 and IM. Risk of drug-related death as a result of diarrhoea is 0.001363473 (section treatment related deaths sum of patients death of diarrhoea/total patients = 19/13935) (162). Therefore, risk of death as a result of grade 3 diarrhoea is 0.1473 x 0.0013 = 0.00020088 for non-actionable *1/*28 and IM. These are given a certainty score of 3.

1.5.3 Absolute risk extraction untested actionables (*28/*28 and PM):

Liu X et al.(125). is a meta-analysis of 16 studies including a total of 2,328 mainly Caucasian patients with colorectal cancer. The outcome measure was grade 3-4 toxicity.

Neutropenia:

Risk of grade 3 or higher neutropenia was 0.3525 (See Figure 2, b, high IRI, sum of events/sum of patients = 43/122) among untested *28/*28 and PM. Risk of drug-related death as a result of myelosupression is 0.00949 (section treatment related deaths: 1.3% died of treatment related effects, of which 73% was associated with myelosuppression) (162). Therefore, risk of death as a result of

grade 3 neutropenia is 0.3525 x 0.00949 = 0.003344836 for untested *28/*28 and PM. These are given a certainty score of 3.

Diarrhoea:

Risk of grade 3 or higher diarrhoea was 0.2155 (See Figure 4, b, high IRI, sum of events/sum of patients = 25/116) among untested *28/*28 and PM. Risk of drug-related death as a result of grade 3 diarrhoea is approximately 0.001363473 (section treatment related deaths sum of patients death of diarrhoea/total patients = 19/13935) (162). Therefore, risk of death as a result of grade 3 diarrhoea is 0.2155 x 0.001 = 0.000293852 for untested *28/*28 and PM. These are given a certainty score of 3.

1.5.4 Absolute risk extraction tested actionables (*28/*28 and PM):

Since no intervention studies were identified, we estimate the risk of death for tested-actionables to equal the risk of death of non-actionables (*1/*1). In this case it is given a certainty score of 0 (estimation).

1.5.5 Conclusion of selected publications and absolute risks extracted (death as a result of neutropenia):

		Actionability	Untested	Ref	CS	Tested	Ref	CS
Irinotecan	UGT1A1 *1/*1	no	0.001064299	(125)	3	0.001064299	(125)	3
Irinotecan	UGT1A1 *1/*28	no	0.001769616	(125)	3	0.001769616	(125)	3
Irinotecan	UGT1A1 *28/*28	yes	0.003344836	(125)	3	0.001064299	-	0
Irinotecan	UGT1A1 IM	no	0.001769616	(125)	3	0.001769616	(125)	3
Irinotecan	UGT1A1 PM	yes	0.003344836	(125)	3	0.001064299	-	0

Ref: Reference; CS: certainty score

1.5.6 Conclusion of selected publications and absolute risks extracted (death as a result of diarrhoea):

		Actionability	Untested	Ref	CS	Tested	Ref	CS
Irinotecan	UGT1A1 *1/*1	no	0.000151267	(125)	3	0.000151267	(125)	3
Irinotecan	UGT1A1 *1/*28	no	0.00020088	(125)	3	0.00020088	(125)	3
Irinotecan	UGT1A1 *28/*28	yes	0.000293852	(125)	3	0.000151267	-	0
Irinotecan	UGT1A1 IM	no	0.00020088	(125)	3	0.00020088	(125)	3
Irinotecan	UGT1A1 PM	yes	0.000293852	(125)	3	0.000151267	-	0

Ref: Reference; CS: certainty score

1.5.7 Conclusion of selected publications and absolute risks extracted (sum absolute risk of death due to neutropenia and absolute risk of death due to diarrhoea):

		Actionability	Untested	Ref	CS	Tested	Ref	CS
Irinotecan	UGT1A1 *1/*1	no	0.001215566	(125)	3	0.001215566	(125)	3
Irinotecan	UGT1A1 *1/*28	no	0.001970496	(125)	3	0.001970496	(125)	3
Irinotecan	UGT1A1 *28/*28	yes	0.003638688	(125)	3	0.001215566	-	0
Irinotecan	UGT1A1 IM	no	0.001970496	(125)	3	0.001970496	(125)	3
Irinotecan	UGT1A1 PM	yes	0.003638688	(125)	3	0.001215566	-	0

Ref: Reference; CS: certainty score

1.6 Assessment of risk of drug-related death following an intermediary outcome associated with the gene-drug interaction

**Interaction**	**Intermediary outcome associated with drug-gene interaction**	**AR of drug-related death as a result of the intermediary outcome**	**Description of reference**	**Ref**
TPMT-azathioprine<br><br>*TPMT*-mercaptopurine<br><br>*TPMT*-thiopurine	Grade≥3 leucopenia	1%	A review of AZA/MP-induced myelotoxicity in inflammatory bowel disease (IBD) patients. In total, 66 studies (8,302 patients) were included. The cumulative incidence of AZA/MP-induced myelotoxicity was 7% (95% confidence interval [CI] 6-8%). The risk of death among patients who developed myelotoxicity was 0.94% (95% CI 0.32-2.70%). The author concludes with: the risk of death among IBD patients who develop myelotoxicity is approximately 1%.	(37)
DPYD-capecitabine<br><br>*DPYD*-fluorouracil	Grade≥3 fluoropyrimidine-induced toxicity	0.75%	This article reviews the pharmacology and efficacy of capecitabine with a special emphasis on its safety. Among seven studies of 290 patients older than 55 years with breast cancer, three treatment-related deaths were observed at the dose of 1255 mg/m2 twice daily on an intermittent schedule (2 weeks on/1 week off).	(75)
CYP2C19-clopidogrel	MACE (death/cardiovascular death, nonfatal myocardial infarction, and nonfatal stroke)*	34%	We calculated the risk of cardiovascular death within MACE from this publication. When multiplied with RR of MACE we are left with risk cardiovascular death. A Cochrane review was used for extraction of this risk. This review regarded clopidogrel plus aspirin versus aspirin alone for preventing cardiovascular events. It includes data from 15 trials with 33,970 people. The risk of cardiovascular death is 37/108 = 0.34 within a median follow-up of 12 months (averages from column Risk with clopidogrel plus aspirin in Summary of findings on page 4 were used).	(120)
UGT1A1-irinotecan	Grade≥3 neutropenia<br><br>Grade≥3 diarrhea	0.9%<br><br>0.1%	A post marketing survey of irinotecan into severe adverse effects and treatment-related deaths. The number of deaths from severe adverse drug reactions<br><br>whose causal relationship with irinotecan could not be ruled<br><br>out was 176 (1.3%) of the 13 935 patients. Of the 176	(162)

			TRDs, 103 (59%) were caused by myelosuppression, 19<br><br>(11%) by myelosuppression accompanied by diarrhea, 6<br><br>(3%) by myelosuppression with ileus, 20 (11%) by interstitial lung disease, 8 (5%) by renal failure, and 1 by diarrhea. Of all TRDs, 73% were associated with myelosuppression, or concurrent incidence of myelosuppression, ileus and diarrhea. Therefore, risk of death as a result of treatment-related myelosuppression is 1.3% * 73% = 0.9% and risk of death as a result of treatment-related diarrhea is 19/13935 = 0.1%.	

1.7 References

(1) Relling, M.V. *et al.* Mercaptopurine therapy intolerance and heterozygosity at the thiopurine S-methyltransferase gene locus. *Journal of the National Cancer Institute* **91**, 2001-8 (1999).

(2) Liu, C. *et al.* Genomewide Approach Validates Thiopurine Methyltransferase Activity Is a Monogenic Pharmacogenomic Trait. *Clinical pharmacology and therapeutics* **101**, 373-81 (2017).

(3) Booth, R.A. *et al.* Assessment of thiopurine S-methyltransferase activity in patients prescribed thiopurines: a systematic review. *Annals of internal medicine* **154**, 814-298 (2011).

(4) Zelinkova, Z. *et al.* Inosine triphosphate pyrophosphatase and thiopurine s-methyltransferase genotypes relationship to azathioprine-induced myelosuppression. *Clinical gastroenterology and hepatology : the official clinical practice journal of the American Gastroenterological Association* **4**, 44-9 (2006).

(5) Fabre, M.A. *et al.* The impact of thiopurine S-methyltransferase polymorphisms on azathioprine dose 1 year after renal transplantation. *Transpl Int* **17**, 531-9 (2004).

(6) Pandya, B., Thomson, W., Poulton, K., Bruce, I., Payne, D. & Qasim, F. Azathioprine toxicity and thiopurine methyltransferase genotype in renal transplant patients. *Transplant Proc* **34**, 1642-5 (2002).

(7) Stanulla, M. *et al.* Thiopurine methyltransferase (TPMT) genotype and early treatment response to mercaptopurine in childhood acute lymphoblastic leukemia. *JAMA* **293**, 1485-9 (2005).

(8) Fan, X., Yin, D., Men, R., Xu, H. & Yang, L. NUDT15 Polymorphism Confer Increased Susceptibility to Thiopurine-Induced Leukopenia in Patients With Autoimmune Hepatitis and Related Cirrhosis. *Frontiers in pharmacology* **10**, 346- (2019).

(9) Choi, R. *et al.* Pathway genes and metabolites in thiopurine therapy in Korean children with acute lymphoblastic leukaemia. *British journal of clinical pharmacology* **85**, 1585-97 (2019).

(10) Eriksen, P.L. *et al.* Enrichment of Genetic Variants in the Glucocorticoid Receptor Signalling Pathway in Autoimmune Hepatitis with Failure of Standard Treatment. *Basic & clinical pharmacology & toxicology* **121**, 189-94 (2017).

(11) Coenen, M.J. *et al.* Identification of Patients With Variants in TPMT and Dose Reduction Reduces Hematologic Events During Thiopurine Treatment of Inflammatory Bowel Disease. *Gastroenterology* **149**, 907-17.e7 (2015).

(12) Lennard, L., Cartwright, C.S., Wade, R. & Vora, A. Thiopurine methyltransferase and treatment outcome in the UK acute lymphoblastic leukaemia trial ALL2003. *Br J Haematol* **170**, 550-8 (2015).

(13) Lennard, L., Cartwright, C.S., Wade, R. & Vora, A. Thiopurine dose intensity and treatment outcome in childhood lymphoblastic leukaemia: the influence of thiopurine methyltransferase pharmacogenetics. *Br J Haematol* **169**, 228-40 (2015).

(14) Kim, M.J., Lee, S.Y. & Choe, Y.H. Monitoring thiopurine metabolites in korean pediatric patients with inflammatory bowel disease. *Yonsei Med J* **55**, 1289-96 (2014).

(15) Levinsen, M. *et al.* Pharmacogenetically based dosing of thiopurines in childhood acute lymphoblastic leukemia: influence on cure rates and risk of second cancer. *Pediatr Blood Cancer* **61**, 797-802 (2014).

(16) Kim, H. *et al.* Pharmacogenetic analysis of pediatric patients with acute lymphoblastic leukemia: a possible association between survival rate and ITPA polymorphism. *PloS one* **7**, e45558-e (2012).

(17) Newman, W.G. *et al.* A pragmatic randomized controlled trial of thiopurine methyltransferase genotyping prior to azathioprine treatment: the TARGET study. *Pharmacogenomics* **12**, 815-26 (2011).
(18) Dong, X.-W., Zheng, Q., Zhu, M.-M., Tong, J.-L. & Ran, Z.-H. Thiopurine S-methyltransferase polymorphisms and thiopurine toxicity in treatment of inflammatory bowel disease. *World J Gastroenterol* **16**, 3187-95 (2010).
(19) Hindorf, U. *et al.* Characterisation and utility of thiopurine methyltransferase and thiopurine metabolite measurements in autoimmune hepatitis. *J Hepatol* **52**, 106-11 (2010).
(20) Sheffield, L.J. *et al.* Thiopurine methyltransferase and thiopurine metabolite testing in patients with inflammatory bowel disease who are taking thiopurine drugs. *Pharmacogenomics* **10**, 1091-9 (2009).
(21) Ansari, A. *et al.* Influence of xanthine oxidase on thiopurine metabolism in Crohn's disease. *Alimentary pharmacology & therapeutics* **28**, 749-57 (2008).
(22) Gardiner, S.J., Gearry, R.B., Begg, E.J., Zhang, M. & Barclay, M.L. Thiopurine dose in intermediate and normal metabolizers of thiopurine methyltransferase may differ three-fold. *Clinical gastroenterology and hepatology : the official clinical practice journal of the American Gastroenterological Association* **6**, 654-04 (2008).
(23) Moloney, F.J., Dicker, P., Conlon, P.J., Shields, D.C. & Murphy, G.M. The frequency and significance of thiopurine S-methyltransferase gene polymorphisms in azathioprine-treated renal transplant recipients. *The British journal of dermatology* **154**, 1199-200 (2006).
(24) Jun, J.B., Cho, D.Y., Kang, C. & Bae, S.C. Thiopurine S-methyltransferase polymorphisms and the relationship between the mutant alleles and the adverse effects in systemic lupus erythematosus patients taking azathioprine. *Clin Exp Rheumatol* **23**, 873-6 (2005).
(25) Stocco, G. *et al.* TPMT genotype and the use of thiopurines in paediatric inflammatory bowel disease. *Dig Liver Dis* **37**, 940-5 (2005).
(26) Kurzawski, M., Dziewanowski, K., Gawrońska-Szklarz, B., Domański, L. & Droździk, M. The impact of thiopurine s-methyltransferase polymorphism on azathioprine-induced myelotoxicity in renal transplant recipients. *Ther Drug Monit* **27**, 435-41 (2005).
(27) Gearry, R.B. *et al.* Thiopurine methyltransferase and 6-thioguanine nucleotide measurement: early experience of use in clinical practice. *Intern Med J* **35**, 580-5 (2005).
(28) Ansari, A. *et al.* Thiopurine methyltransferase activity and the use of azathioprine in inflammatory bowel disease. *Alimentary pharmacology & therapeutics* **16**, 1743-50 (2002).
(29) Langley, P.G., Underhill, J., Tredger, J.M., Norris, S. & McFarlane, I.G. Thiopurine methyltransferase phenotype and genotype in relation to azathioprine therapy in autoimmune hepatitis. *J Hepatol* **37**, 441-7 (2002).
(30) Regueiro, M. & Mardini, H. Determination of thiopurine methyltransferase genotype or phenotype optimizes initial dosing of azathioprine for the treatment of Crohn's disease. *J Clin Gastroenterol* **35**, 240-4 (2002).
(31) Campbell, S., Kingstone, K. & Ghosh, S. Relevance of thiopurine methyltransferase activity in inflammatory bowel disease patients maintained on low-dose azathioprine. *Alimentary pharmacology & therapeutics* **16**, 389-98 (2002).
(32) Colombel, J.F. *et al.* Genotypic analysis of thiopurine S-methyltransferase in patients with Crohn's disease and severe myelosuppression during azathioprine therapy. *Gastroenterology* **118**, 1025-30 (2000).
(33) Black, A.J. *et al.* Thiopurine methyltransferase genotype predicts therapy-limiting severe toxicity from azathioprine. *Annals of internal medicine* **129**, 716-8 (1998).

(34) Higgs, J.E., Payne, K., Roberts, C. & Newman, W.G. Are patients with intermediate TPMT activity at increased risk of myelosuppression when taking thiopurine medications? *Pharmacogenomics* **11**, 177-88 (2010).
(35) Evans, W.E. *et al.* Preponderance of thiopurine S-methyltransferase deficiency and heterozygosity among patients intolerant to mercaptopurine or azathioprine. *Journal of clinical oncology : official journal of the American Society of Clinical Oncology* **19**, 2293-301 (2001).
(36) McLeod, H.L. *et al.* Analysis of thiopurine methyltransferase variant alleles in childhood acute lymphoblastic leukaemia. *Br J Haematol* **105**, 696-700 (1999).
(37) Gisbert, J.P. & Gomollón, F. Thiopurine-induced myelotoxicity in patients with inflammatory bowel disease: a review. *Am J Gastroenterol* **103**, 1783-800 (2008).
(38) Wray, L. *et al.* TPMT and MTHFR genotype is not associated with altered risk of thioguanine-related sinusoidal obstruction syndrome in pediatric acute lymphoblastic leukemia: a report from the Children's Oncology Group. *Pediatr Blood Cancer* **61**, 2086-8 (2014).
(39) Lennard, L. *et al.* The thiopurine methyltransferase genetic polymorphism is associated with thioguanine-related veno-occlusive disease of the liver in children with acute lymphoblastic leukemia. *Clinical pharmacology and therapeutics* **80**, 375-83 (2006).
(40) Teml, A. *et al.* A prospective, open-label trial of 6-thioguanine in patients with ulcerative or indeterminate colitis. *Scand J Gastroenterol* **40**, 1205-13 (2005).
(41) Herrlinger, K.R. *et al.* Thioguanine-nucleotides do not predict efficacy of tioguanine in Crohn's disease. *Alimentary pharmacology & therapeutics* **19**, 1269-76 (2004).
(42) Deenen, M.J. *et al.* Relationship between single nucleotide polymorphisms and haplotypes in DPYD and toxicity and efficacy of capecitabine in advanced colorectal cancer. *Clinical cancer research : an official journal of the American Association for Cancer Research* **17**, 3455-68 (2011).
(43) Meulendijks, D. *et al.* Pretreatment serum uracil concentration as a predictor of severe and fatal fluoropyrimidine-associated toxicity. *Br J Cancer* **116**, 1415-24 (2017).
(44) Meulendijks, D. *et al.* Clinical relevance of DPYD variants c.1679T>G, c.1236G>A/HapB3, and c.1601G>A as predictors of severe fluoropyrimidine-associated toxicity: a systematic review and meta-analysis of individual patient data. *The Lancet Oncology* **16**, 1639-50 (2015).
(45) Rosmarin, D. *et al.* Genetic markers of toxicity from capecitabine and other fluorouracil-based regimens: investigation in the QUASAR2 study, systematic review, and meta-analysis. *Journal of clinical oncology : official journal of the American Society of Clinical Oncology* **32**, 1031-9 (2014).
(46) Terrazzino, S., Cargnin, S., Del Re, M., Danesi, R., Canonico, P.L. & Genazzani, A.A. DPYD IVS14+1G>A and 2846A>T genotyping for the prediction of severe fluoropyrimidine-related toxicity: a meta-analysis. *Pharmacogenomics* **14**, 1255-72 (2013).
(47) Vulsteke, C. *et al.* Genetic variability in the multidrug resistance associated protein-1 (ABCC1/MRP1) predicts hematological toxicity in breast cancer patients receiving (neo-)adjuvant chemotherapy with 5-fluorouracil, epirubicin and cyclophosphamide (FEC). *Ann Oncol* **24**, 1513-25 (2013).
(48) Kleinjan, J.P., Brinkman, I., Bakema, R., van Zanden, J.J. & van Rooijen, J.M. Tolerance-based capecitabine dose escalation after DPYD genotype-guided dosing in heterozygote DPYD variant carriers: a single-center observational study. *Anticancer Drugs* **30**, 410-5 (2019).

(49) Henricks, L.M. *et al.* Effectiveness and safety of reduced-dose fluoropyrimidine therapy in patients carrying the DPYD*2A variant: A matched pair analysis. *Int J Cancer* **144**, 2347-54 (2019).

(50) Lunenburg, C.A.T.C. *et al.* Standard fluoropyrimidine dosages in chemoradiation therapy result in an increased risk of severe toxicity in DPYD variant allele carriers. *European journal of cancer (Oxford, England : 1990)* **104**, 210-8 (2018).

(51) Henricks, L.M. *et al.* DPYD genotype-guided dose individualisation of fluoropyrimidine therapy in patients with cancer: a prospective safety analysis. *The Lancet Oncology* **19**, 1459-67 (2018).

(52) Madi, A. *et al.* Pharmacogenetic analyses of 2183 patients with advanced colorectal cancer; potential role for common dihydropyrimidine dehydrogenase variants in toxicity to chemotherapy. *European journal of cancer (Oxford, England : 1990)* **102**, 31-9 (2018).

(53) Lunenburg, C.A., van Staveren, M.C., Gelderblom, H., Guchelaar, H.J. & Swen, J.J. Evaluation of clinical implementation of prospective DPYD genotyping in 5-fluorouracil- or capecitabine-treated patients. *Pharmacogenomics* **17**, 721-9 (2016).

(54) Lee, A.M. *et al.* Association between DPYD c.1129-5923 C>G/hapB3 and severe toxicity to 5-fluorouracil-based chemotherapy in stage III colon cancer patients: NCCTG N0147 (Alliance). *Pharmacogenetics and genomics* **26**, 133-7 (2016).

(55) Deenen, M.J. *et al.* Upfront Genotyping of DPYD*2A to Individualize Fluoropyrimidine Therapy: A Safety and Cost Analysis. *Journal of clinical oncology : official journal of the American Society of Clinical Oncology* **34**, 227-34 (2016).

(56) Lee, A.M. *et al.* DPYD variants as predictors of 5-fluorouracil toxicity in adjuvant colon cancer treatment (NCCTG N0147). *Journal of the National Cancer Institute* **106**, dju298 (2014).

(57) van Kuilenburg, A.B.P. *et al.* Evaluation of 5-fluorouracil pharmacokinetics in cancer patients with a c.1905+1G>A mutation in DPYD by means of a Bayesian limited sampling strategy. *Clinical pharmacokinetics* **51**, 163-74 (2012).

(58) Kristensen, M.H., Pedersen, P.L., Melsen, G.V., Ellehauge, J. & Mejer, J. Variants in the dihydropyrimidine dehydrogenase, methylenetetrahydrofolate reductase and thymidylate synthase genes predict early toxicity of 5-fluorouracil in colorectal cancer patients. *J Int Med Res* **38**, 870-83 (2010).

(59) Gross, E. *et al.* Strong association of a common dihydropyrimidine dehydrogenase gene polymorphism with fluoropyrimidine-related toxicity in cancer patients. *PloS one* **3**, e4003-e (2008).

(60) Capitain, O., Boisdron-Celle, M., Poirier, A.L., Abadie-Lacourtoisie, S., Morel, A. & Gamelin, E. The influence of fluorouracil outcome parameters on tolerance and efficacy in patients with advanced colorectal cancer. *The pharmacogenomics journal* **8**, 256-67 (2008).

(61) Sulzyc-Bielicka, V. *et al.* 5-Fluorouracil toxicity-attributable IVS14 + 1G > A mutation of the dihydropyrimidine dehydrogenase gene in Polish colorectal cancer patients. *Pharmacol Rep* **60**, 238-42 (2008).

(62) Schwab, M. *et al.* Role of genetic and nongenetic factors for fluorouracil treatment-related severe toxicity: a prospective clinical trial by the German 5-FU Toxicity Study Group. *Journal of clinical oncology : official journal of the American Society of Clinical Oncology* **26**, 2131-8 (2008).

(63) Jatoi, A. *et al.* Paclitaxel, carboplatin, 5-fluorouracil, and radiation for locally advanced esophageal cancer: phase II results of preliminary pharmacologic and molecular efforts to

mitigate toxicity and predict outcomes: North Central Cancer Treatment Group (N0044). *Am J Clin Oncol* **30**, 507-13 (2007).
(64) Magné, N. *et al.* Dihydropyrimidine dehydrogenase activity and the IVS14+1G>A mutation in patients developing 5FU-related toxicity. *British journal of clinical pharmacology* **64**, 237-40 (2007).
(65) Boisdron-Celle, M. *et al.* 5-Fluorouracil-related severe toxicity: a comparison of different methods for the pretherapeutic detection of dihydropyrimidine dehydrogenase deficiency. *Cancer Lett* **249**, 271-82 (2007).
(66) Cho, H.-J., Park, Y.S., Kang, W.K., Kim, J.-W. & Lee, S.-Y. Thymidylate synthase (TYMS) and dihydropyrimidine dehydrogenase (DPYD) polymorphisms in the Korean population for prediction of 5-fluorouracil-associated toxicity. *Ther Drug Monit* **29**, 190-6 (2007).
(67) Salgado, J., Zabalegui, N., Gil, C., Monreal, I., Rodríguez, J. & García-Foncillas, J. Polymorphisms in the thymidylate synthase and dihydropyrimidine dehydrogenase genes predict response and toxicity to capecitabine-raltitrexed in colorectal cancer. *Oncol Rep* **17**, 325-8 (2007).
(68) Morel, A. *et al.* Clinical relevance of different dihydropyrimidine dehydrogenase gene single nucleotide polymorphisms on 5-fluorouracil tolerance. *Mol Cancer Ther* **5**, 2895-904 (2006).
(69) Largillier, R. *et al.* Pharmacogenetics of capecitabine in advanced breast cancer patients. *Clinical cancer research : an official journal of the American Association for Cancer Research* **12**, 5496-502 (2006).
(70) Salgueiro, N. *et al.* Mutations in exon 14 of dihydropyrimidine dehydrogenase and 5-Fluorouracil toxicity in Portuguese colorectal cancer patients. *Genetics in medicine : official journal of the American College of Medical Genetics* **6**, 102-7 (2004).
(71) Van Kuilenburg, A.B., Meinsma, R., Zoetekouw, L. & Van Gennip, A.H. High prevalence of the IVS14 + 1G>A mutation in the dihydropyrimidine dehydrogenase gene of patients with severe 5-fluorouracil-associated toxicity. *Pharmacogenetics* **12**, 555-8 (2002).
(72) Raida, M. *et al.* Prevalence of a common point mutation in the dihydropyrimidine dehydrogenase (DPD) gene within the 5'-splice donor site of intron 14 in patients with severe 5-fluorouracil (5-FU)- related toxicity compared with controls. *Clinical cancer research : an official journal of the American Association for Cancer Research* **7**, 2832-9 (2001).
(73) Yamaguchi, K., Arai, Y., Kanda, Y. & Akagi, K. Germline mutation of dihydropyrimidine dehydrogenese gene among a Japanese population in relation to toxicity to 5-Fluorouracil. *Jpn J Cancer Res* **92**, 337-42 (2001).
(74) van Kuilenburg, A.B. *et al.* Clinical implications of dihydropyrimidine dehydrogenase (DPD) deficiency in patients with severe 5-fluorouracil-associated toxicity: identification of new mutations in the DPD gene. *Clinical cancer research : an official journal of the American Association for Cancer Research* **6**, 4705-12 (2000).
(75) Mikhail, S.E., Sun, J.F. & Marshall, J.L. Safety of capecitabine: a review. *Expert opinion on drug safety* **9**, 831-41 (2010).
(76) Niu, X. *et al.* CYP2C19 polymorphism and clinical outcomes among patients of different races treated with clopidogrel: A systematic review and meta-analysis. *J Huazhong Univ Sci Technolog Med Sci* **35**, 147-56 (2015).
(77) Jang, J.-S. *et al.* Meta-analysis of cytochrome P450 2C19 polymorphism and risk of adverse clinical outcomes among coronary artery disease patients of different ethnic groups treated with clopidogrel. *Am J Cardiol* **110**, 502-8 (2012).
(78) Pan, Y. *et al.* Genetic Polymorphisms and Clopidogrel Efficacy for Acute Ischemic Stroke or Transient Ischemic Attack: A Systematic Review and Meta-Analysis. *Circulation* **135**, 21-33 (2017).

(79) Sorich, M.J., Rowland, A., McKinnon, R.A. & Wiese, M.D. CYP2C19 genotype has a greater effect on adverse cardiovascular outcomes following percutaneous coronary intervention and in Asian populations treated with clopidogrel: a meta-analysis. *Circ Cardiovasc Genet* **7**, 895-902 (2014).

(80) Mao, L. *et al.* Cytochrome CYP2C19 polymorphism and risk of adverse clinical events in clopidogrel-treated patients: a meta-analysis based on 23,035 subjects. *Arch Cardiovasc Dis* **106**, 517-27 (2013).

(81) Li, Y., Tang, H.L., Hu, Y.F. & Xie, H.G. The gain-of-function variant allele CYP2C19*17: a double-edged sword between thrombosis and bleeding in clopidogrel-treated patients. *J Thromb Haemost* **10**, 199-206 (2012).

(82) Holmes, M.V., Perel, P., Shah, T., Hingorani, A.D. & Casas, J.P. CYP2C19 genotype, clopidogrel metabolism, platelet function, and cardiovascular events: a systematic review and meta-analysis. *JAMA* **306**, 2704-14 (2011).

(83) Liu, Y.P. *et al.* Association of genetic variants in CYP2C19 and adverse clinical outcomes after treatment with clopidogrel: an updated meta-analysis. *Thromb Res* **128**, 593-4 (2011).

(84) Mega, J.L. *et al.* Dosing clopidogrel based on CYP2C19 genotype and the effect on platelet reactivity in patients with stable cardiovascular disease. *JAMA* **306**, 2221-8 (2011).

(85) Simon, T. *et al.* Genetic polymorphisms and the impact of a higher clopidogrel dose regimen on active metabolite exposure and antiplatelet response in healthy subjects. *Clinical pharmacology and therapeutics* **90**, 287-95 (2011).

(86) Collet, J.-P. *et al.* Cytochrome P450 2C19 polymorphism in young patients treated with clopidogrel after myocardial infarction: a cohort study. *Lancet (London, England)* **373**, 309-17 (2009).

(87) Simon, T. *et al.* Genetic determinants of response to clopidogrel and cardiovascular events. *The New England journal of medicine* **360**, 363-75 (2009).

(88) Shen, D.-L. *et al.* Clinical Value of CYP2C19 Genetic Testing for Guiding the Antiplatelet Therapy in a Chinese Population. *J Cardiovasc Pharmacol* **67**, 232-6 (2016).

(89) Mega, J.L. *et al.* Cytochrome p-450 polymorphisms and response to clopidogrel. *The New England journal of medicine* **360**, 354-62 (2009).

(90) Geisler, T. *et al.* CYP2C19 and nongenetic factors predict poor responsiveness to clopidogrel loading dose after coronary stent implantation. *Pharmacogenomics* **9**, 1251-9 (2008).

(91) Chen, B.-L. *et al.* Inhibition of ADP-induced platelet aggregation by clopidogrel is related to CYP2C19 genetic polymorphisms. *Clinical and experimental pharmacology & physiology* **35**, 904-8 (2008).

(92) Kim, K.A., Park, P.W., Hong, S.J. & Park, J.Y. The effect of CYP2C19 polymorphism on the pharmacokinetics and pharmacodynamics of clopidogrel: a possible mechanism for clopidogrel resistance. *Clinical pharmacology and therapeutics* **84**, 236-42 (2008).

(93) Malek, L.A. *et al.* Coexisting polymorphisms of P2Y12 and CYP2C19 genes as a risk factor for persistent platelet activation with clopidogrel. *Circ J* **72**, 1165-9 (2008).

(94) Trenk, D. *et al.* Cytochrome P450 2C19 681G>A polymorphism and high on-clopidogrel platelet reactivity associated with adverse 1-year clinical outcome of elective percutaneous coronary intervention with drug-eluting or bare-metal stents. *J Am Coll Cardiol* **51**, 1925-34 (2008).

(95) Fontana, P., Hulot, J.S., De Moerloose, P. & Gaussem, P. Influence of CYP2C19 and CYP3A4 gene polymorphisms on clopidogrel responsiveness in healthy subjects. *J Thromb Haemost* **5**, 2153-5 (2007).

(96) Hulot, J.-S. *et al.* Cytochrome P450 2C19 loss-of-function polymorphism is a major determinant of clopidogrel responsiveness in healthy subjects. *Blood* **108**, 2244-7 (2006).
(97) Lee, C.R. *et al.* Clinical Outcomes and Sustainability of Using CYP2C19 Genotype-Guided Antiplatelet Therapy After Percutaneous Coronary Intervention. *Circ Genom Precis Med* **11**, e002069-e (2018).
(98) Zhong, Z. *et al.* Effect of cytochrome P450 2C19 polymorphism on adverse cardiovascular events after drug-eluting stent implantation in a large Hakka population with acute coronary syndrome receiving clopidogrel in southern China. *European journal of clinical pharmacology* **74**, 423-31 (2018).
(99) Wu, Y. *et al.* Impact of CYP2C19 polymorphism in prognosis of minor stroke or TIA patients with declined eGFR on dual antiplatelet therapy: CHANCE substudy. *The pharmacogenomics journal* **18**, 713-20 (2018).
(100) Cavallari, L.H. *et al.* Multisite Investigation of Outcomes With Implementation of CYP2C19 Genotype-Guided Antiplatelet Therapy After Percutaneous Coronary Intervention. *JACC Cardiovasc Interv* **11**, 181-91 (2018).
(101) Lin, Y. *et al.* Impact of Glycemic Control on Efficacy of Clopidogrel in Transient Ischemic Attack or Minor Stroke Patients With CYP2C19 Genetic Variants. *Stroke* **48**, 998-1004 (2017).
(102) Deiman, B.A.L.M. *et al.* Reduced number of cardiovascular events and increased cost-effectiveness by genotype-guided antiplatelet therapy in patients undergoing percutaneous coronary interventions in the Netherlands. *Neth Heart J* **24**, 589-99 (2016).
(103) Wang, Y. *et al.* Association Between CYP2C19 Loss-of-Function Allele Status and Efficacy of Clopidogrel for Risk Reduction Among Patients With Minor Stroke or Transient Ischemic Attack. *JAMA* **316**, 70-8 (2016).
(104) Ogawa, H. *et al.* Effects of CYP2C19 allelic variants on inhibition of platelet aggregation and major adverse cardiovascular events in Japanese patients with acute coronary syndrome: The PRASFIT-ACS study. *J Cardiol* **68**, 29-36 (2016).
(105) Xiong, R., Liu, W., Chen, L., Kang, T., Ning, S. & Li, J. A randomized controlled trial to assess the efficacy and safety of doubling dose clopidogrel versus ticagrelor for the treatment of acute coronary syndrome in patients with CYP2C19*2 homozygotes. *Int J Clin Exp Med* **8**, 13310-6 (2015).
(106) Xie, X. *et al.* Personalized antiplatelet therapy according to CYP2C19 genotype after percutaneous coronary intervention: a randomized control trial. *Int J Cardiol* **168**, 3736-40 (2013).
(107) Collet, J.-P. *et al.* High doses of clopidogrel to overcome genetic resistance: the randomized crossover CLOVIS-2 (Clopidogrel and Response Variability Investigation Study 2). *JACC Cardiovasc Interv* **4**, 392-402 (2011).
(108) Bonello-Palot, N. *et al.* Relation of body mass index to high on-treatment platelet reactivity and of failed clopidogrel dose adjustment according to platelet reactivity monitoring in patients undergoing percutaneous coronary intervention. *Am J Cardiol* **104**, 1511-5 (2009).
(109) Shuldiner, A.R. *et al.* Association of cytochrome P450 2C19 genotype with the antiplatelet effect and clinical efficacy of clopidogrel therapy. *JAMA* **302**, 849-57 (2009).
(110) Frére, C., Cuisset, T., Gaborit, B., Alessi, M.C. & Hulot, J.S. The CYP2C19*17 allele is associated with better platelet response to clopidogrel in patients admitted for non-ST acute coronary syndrome. *J Thromb Haemost* **7**, 1409-11 (2009).
(111) Aleil, B., Léon, C., Cazenave, J.P. & Gachet, C. CYP2C19*2 polymorphism is not the sole determinant of the response to clopidogrel: implications for its monitoring. *J Thromb Haemost* **7**, 1747-9 (2009).

(112) Sibbing, D. *et al.* Cytochrome P450 2C19 loss-of-function polymorphism and stent thrombosis following percutaneous coronary intervention. *Eur Heart J* **30**, 916-22 (2009).

(113) Brackbill, M.L., Kidd, R.S., Abdoo, A.D., Warner, J.G., Jr. & Harralson, A.F. Frequency of CYP3A4, CYP3A5, CYP2C9, and CYP2C19 variant alleles in patients receiving clopidogrel that experience repeat acute coronary syndrome. *Heart Vessels* **24**, 73-8 (2009).

(114) Giusti, B. *et al.* Relation of cytochrome P450 2C19 loss-of-function polymorphism to occurrence of drug-eluting coronary stent thrombosis. *Am J Cardiol* **103**, 806-11 (2009).

(115) Umemura, K., Furuta, T. & Kondo, K. The common gene variants of CYP2C19 affect pharmacokinetics and pharmacodynamics in an active metabolite of clopidogrel in healthy subjects. *J Thromb Haemost* **6**, 1439-41 (2008).

(116) Frere, C. *et al.* Effect of cytochrome p450 polymorphisms on platelet reactivity after treatment with clopidogrel in acute coronary syndrome. *Am J Cardiol* **101**, 1088-93 (2008).

(117) Fontana, P., Senouf, D. & Mach, F. Biological effect of increased maintenance dose of clopidogrel in cardiovascular outpatients and influence of the cytochrome P450 2C19*2 allele on clopidogrel responsiveness. *Thromb Res* **121**, 463-8 (2008).

(118) Giusti, B. *et al.* Cytochrome P450 2C19 loss-of-function polymorphism, but not CYP3A4 IVS10 + 12G/A and P2Y12 T744C polymorphisms, is associated with response variability to dual antiplatelet treatment in high-risk vascular patients. *Pharmacogenetics and genomics* **17**, 1057-64 (2007).

(119) Brandt, J.T. *et al.* Common polymorphisms of CYP2C19 and CYP2C9 affect the pharmacokinetic and pharmacodynamic response to clopidogrel but not prasugrel. *J Thromb Haemost* **5**, 2429-36 (2007).

(120) Squizzato, A., Bellesini, M., Takeda, A., Middeldorp, S. & Donadini, M.P. Clopidogrel plus aspirin versus aspirin alone for preventing cardiovascular events. *Cochrane Database of Systematic Reviews*, (2017).

(121) Chen, X. *et al.* UGT1A1 polymorphisms with irinotecan-induced toxicities and treatment outcome in Asians with Lung Cancer: a meta-analysis. *Cancer Chemother Pharmacol* **79**, 1109-17 (2017).

(122) Liu, X.-H. *et al.* Predictive Value of UGT1A1*28 Polymorphism In Irinotecan-based Chemotherapy. *J Cancer* **8**, 691-703 (2017).

(123) Han, F.-f. *et al.* Associations between UGT1A1*6 or UGT1A1*6/*28 polymorphisms and irinotecan-induced neutropenia in Asian cancer patients. *Cancer Chemother Pharmacol* **73**, 779-88 (2014).

(124) Chen, Y.-J. *et al.* The association of UGT1A1*6 and UGT1A1*28 with irinotecan-induced neutropenia in Asians: a meta-analysis. *Biomarkers* **19**, 56-62 (2014).

(125) Liu, X., Cheng, D., Kuang, Q., Liu, G. & Xu, W. Association of UGT1A1*28 polymorphisms with irinotecan-induced toxicities in colorectal cancer: a meta-analysis in Caucasians. *The pharmacogenomics journal* **14**, 120-9 (2014).

(126) Hu, Z.-Y., Yu, Q., Pei, Q. & Guo, C. Dose-dependent association between UGT1A1*28 genotype and irinotecan-induced neutropenia: low doses also increase risk. *Clinical cancer research : an official journal of the American Association for Cancer Research* **16**, 3832-42 (2010).

(127) Hu, Z.-Y., Yu, Q. & Zhao, Y.-S. Dose-dependent association between UGT1A1*28 polymorphism and irinotecan-induced diarrhoea: a meta-analysis. *European journal of cancer (Oxford, England : 1990)* **46**, 1856-65 (2010).

(128) Hoskins, J.M., Goldberg, R.M., Qu, P., Ibrahim, J.G. & McLeod, H.L. UGT1A1*28 genotype and irinotecan-induced neutropenia: dose matters. *Journal of the National Cancer Institute* **99**, 1290-5 (2007).
(129) Dias, M.M. *et al.* The effect of the UGT1A1*28 allele on survival after irinotecan-based chemotherapy: a collaborative meta-analysis. *The pharmacogenomics journal* **14**, 424-31 (2014).
(130) Liu, X., Cheng, D., Kuang, Q., Liu, G. & Xu, W. Association between UGT1A1*28 polymorphisms and clinical outcomes of irinotecan-based chemotherapies in colorectal cancer: a meta-analysis in Caucasians. *PloS one* **8**, e58489-e (2013).
(131) Dias, M.M., McKinnon, R.A. & Sorich, M.J. Impact of the UGT1A1*28 allele on response to irinotecan: a systematic review and meta-analysis. *Pharmacogenomics* **13**, 889-99 (2012).
(132) Denlinger, C.S. *et al.* Pharmacokinetic analysis of irinotecan plus bevacizumab in patients with advanced solid tumors. *Cancer Chemother Pharmacol* **65**, 97-105 (2009).
(133) Lu, C.-Y. *et al.* Clinical Implication of UGT1A1 Promoter Polymorphism for Irinotecan Dose Escalation in Metastatic Colorectal Cancer Patients Treated with Bevacizumab Combined with FOLFIRI in the First-line Setting. *Transl Oncol* **8**, 474-9 (2015).
(134) Goetz, M.P. *et al.* UGT1A1 genotype-guided phase I study of irinotecan, oxaliplatin, and capecitabine. *Invest New Drugs* **31**, 1559-67 (2013).
(135) Kweekel, D.M. *et al.* UGT1A1*28 genotype and irinotecan dosage in patients with metastatic colorectal cancer: a Dutch Colorectal Cancer Group study. *Br J Cancer* **99**, 275-82 (2008).
(136) Liu, C.-Y. *et al.* UGT1A1*28 polymorphism predicts irinotecan-induced severe toxicities without affecting treatment outcome and survival in patients with metastatic colorectal carcinoma. *Cancer* **112**, 1932-40 (2008).
(137) Lankisch, T.O. *et al.* Gilbert's Syndrome and irinotecan toxicity: combination with UDP-glucuronosyltransferase 1A7 variants increases risk. *Cancer Epidemiol Biomarkers Prev* **17**, 695-701 (2008).
(138) Minami, H. *et al.* Irinotecan pharmacokinetics/pharmacodynamics and UGT1A genetic polymorphisms in Japanese: roles of UGT1A1*6 and *28. *Pharmacogenetics and genomics* **17**, 497-504 (2007).
(139) Stewart, C.F. *et al.* UGT1A1 promoter genotype correlates with SN-38 pharmacokinetics, but not severe toxicity in patients receiving low-dose irinotecan. *Journal of clinical oncology : official journal of the American Society of Clinical Oncology* **25**, 2594-600 (2007).
(140) Côté, J.-F. *et al.* UGT1A1 polymorphism can predict hematologic toxicity in patients treated with irinotecan. *Clinical cancer research : an official journal of the American Association for Cancer Research* **13**, 3269-75 (2007).
(141) Ramchandani, R.P. *et al.* The role of SN-38 exposure, UGT1A1*28 polymorphism, and baseline bilirubin level in predicting severe irinotecan toxicity. *Journal of clinical pharmacology* **47**, 78-86 (2007).
(142) Romero, R.Z. *et al.* Potential application of GSTT1-null genotype in predicting toxicity associated to 5-fluouracil irinotecan and leucovorin regimen in advanced stage colorectal cancer patients. *Oncol Rep* **16**, 497-503 (2006).
(143) de Jong, F.A. *et al.* Prophylaxis of irinotecan-induced diarrhea with neomycin and potential role for UGT1A1*28 genotype screening: a double-blind, randomized, placebo-controlled study. *The oncologist* **11**, 944-54 (2006).
(144) Toffoli, G. *et al.* The role of UGT1A1*28 polymorphism in the pharmacodynamics and pharmacokinetics of irinotecan in patients with metastatic colorectal cancer. *Journal of clinical oncology : official journal of the American Society of Clinical Oncology* **24**, 3061-8 (2006).

(145) Han, J.-Y. *et al.* Comprehensive analysis of UGT1A polymorphisms predictive for pharmacokinetics and treatment outcome in patients with non-small-cell lung cancer treated with irinotecan and cisplatin. *Journal of clinical oncology : official journal of the American Society of Clinical Oncology* **24**, 2237-44 (2006).

(146) McLeod, H.L. *et al.* Pharmacogenetic predictors of adverse events and response to chemotherapy in metastatic colorectal cancer: results from North American Gastrointestinal Intergroup Trial N9741. *Journal of clinical oncology : official journal of the American Society of Clinical Oncology* **28**, 3227-33 (2010).

(147) Massacesi, C. *et al.* Uridine diphosphate glucuronosyl transferase 1A1 promoter polymorphism predicts the risk of gastrointestinal toxicity and fatigue induced by irinotecan-based chemotherapy. *Cancer* **106**, 1007-16 (2006).

(148) Wright, M.A. *et al.* A phase I pharmacologic and pharmacogenetic trial of sequential 24-hour infusion of irinotecan followed by leucovorin and a 48-hour infusion of fluorouracil in adult patients with solid tumors. *Clinical cancer research : an official journal of the American Association for Cancer Research* **11**, 4144-50 (2005).

(149) Soepenberg, O. *et al.* Phase I pharmacokinetic, food effect, and pharmacogenetic study of oral irinotecan given as semisolid matrix capsules in patients with solid tumors. *Clinical cancer research : an official journal of the American Association for Cancer Research* **11**, 1504-11 (2005).

(150) Zhou, Q. *et al.* Pharmacogenetic profiling across the irinotecan pathway in Asian patients with cancer. *British journal of clinical pharmacology* **59**, 415-24 (2005).

(151) Carlini, L.E. *et al.* UGT1A7 and UGT1A9 polymorphisms predict response and toxicity in colorectal cancer patients treated with capecitabine/irinotecan. *Clinical cancer research : an official journal of the American Association for Cancer Research* **11**, 1226-36 (2005).

(152) Kitagawa, C. *et al.* Genetic polymorphism in the phenobarbital-responsive enhancer module of the UDP-glucuronosyltransferase 1A1 gene and irinotecan toxicity. *Pharmacogenetics and genomics* **15**, 35-41 (2005).

(153) Marcuello, E., Altés, A., Menoyo, A., Del Rio, E., Gómez-Pardo, M. & Baiget, M. UGT1A1 gene variations and irinotecan treatment in patients with metastatic colorectal cancer. *Br J Cancer* **91**, 678-82 (2004).

(154) Rouits, E., Boisdron-Celle, M., Dumont, A., Guérin, O., Morel, A. & Gamelin, E. Relevance of different UGT1A1 polymorphisms in irinotecan-induced toxicity: a molecular and clinical study of 75 patients. *Clinical cancer research : an official journal of the American Association for Cancer Research* **10**, 5151-9 (2004).

(155) Paoluzzi, L. *et al.* Influence of genetic variants in UGT1A1 and UGT1A9 on the in vivo glucuronidation of SN-38. *Journal of clinical pharmacology* **44**, 854-60 (2004).

(156) Sai, K. *et al.* UGT1A1 haplotypes associated with reduced glucuronidation and increased serum bilirubin in irinotecan-administered Japanese patients with cancer. *Clinical pharmacology and therapeutics* **75**, 501-15 (2004).

(157) Innocenti, F. *et al.* Genetic variants in the UDP-glucuronosyltransferase 1A1 gene predict the risk of severe neutropenia of irinotecan. *Journal of clinical oncology : official journal of the American Society of Clinical Oncology* **22**, 1382-8 (2004).

(158) Font, A. *et al.* Weekly regimen of irinotecan/docetaxel in previously treated non-small cell lung cancer patients and correlation with uridine diphosphate glucuronosyltransferase 1A1 (UGT1A1) polymorphism. *Invest New Drugs* **21**, 435-43 (2003).

(159) Mathijssen, R.H.J. *et al.* Irinotecan pathway genotype analysis to predict pharmacokinetics. *Clinical cancer research : an official journal of the American Association for Cancer Research* **9**, 3246-53 (2003).

(160) Iyer, L. *et al.* UGT1A1*28 polymorphism as a determinant of irinotecan disposition and toxicity. *The pharmacogenomics journal* **2**, 43-7 (2002).

(161) Ando, Y. *et al.* Polymorphisms of UDP-glucuronosyltransferase gene and irinotecan toxicity: a pharmacogenetic analysis. *Cancer Res* **60**, 6921-6 (2000).

(162) Shiozawa, T. *et al.* Risk factors for severe adverse effects and treatment-related deaths in Japanese patients treated with irinotecan-based chemotherapy: a postmarketing survey. *Jpn J Clin Oncol* **43**, 483-91 (2013).

Supplementary Table 4 Costs used in the decision analytic model

Input	Regimen	Dose form	Cost	Source
PGx test for *TMPT*	-	-	€ 132 per test	Leiden University Medical Center prices based on the Dutch Healthcare Authority (NZa) in 2018
PGx test for *DPYD*	-	-	€ 132 per test	Leiden University Medical Center prices based on the Dutch Healthcare Authority (NZa) in 2018
PGx test for *CYP2C19*	-	-	€ 132 per test	Leiden University Medical Center prices based on the Dutch Healthcare Authority (NZa) in 2018
PGx test for *UGT1A1*	-	-	€ 66 per test	Leiden University Medical Center prices based on the Dutch Healthcare Authority (NZa) in 2018
Pharmacist time	-	-	€12.11 per 18 minutes	Time: Ref van der wouden et al. (*unpublished*) (1)<br><br>Salary: Clinical Pharmacists as standardized in Dutch Academic Hospitals in 2019 (2)
Physician time	-	-	€4.28 per 6 minutes	Time: Estimation based on Ref. (*unpublished*) (1)<br><br>Salary: Medical Specialists as standardized in Dutch Academic Hospitals in 2019 (2)
Azathioprine 100% (EM)	1dd 2mg/kg	2x tablet 75mg	€ 0.34 per tablet	Medicijnkosten.nl (3)
Azathioprine 50% (IM)	1dd 1mg/kg	2 x tablet 75mg	€ 0.34 per tablet	Medicijnkosten.nl (3)
Azathioprine 10% (PM)	1dd 0.5mg/kg	1 x tablet 50mg	€ 0.19 per tablet	Medicijnkosten.nl (3)
Capecitabine 100% (GAS 2)	2dd 1250mg/m^2 for 2 weeks. 1	4 x tablet 500mg	€ 1.24 per tablet	Medicijnkosten.nl (3)

	week rest. for 6 months			
Capecitabine 50% (GAS 1.5, 1)	2dd 625mg/m^2 for 2 weeks. 1 week rest. for 6 months	2x tablet 500mg	€ 1.24 per tablet	Medicijnkosten.nl (3)
Capecitabine alternative (GAS 0.5, 0)	Assumed same cost as capecitabine 100%			
Clopidogrel (EM, UM)	1dd75mg	1x tablet 75mg	€ 0.04 per tablet	Medicijnkosten.nl (3)
Clopidogrel 200% (IM)	1dd150mg	2x tablet 75mg	€ 0.04 per tablet	Medicijnkosten.nl (3)
Clopidogrel alternative 1 (PM, ACS -25%)	ticagrelor: 2dd90mg	2 x tablet 90mg	€ 1.24 per tablet	Medicijnkosten.nl (3)
Clopidogrel alternative 2 (PM, ACS - 25%)	prasugrel: 1dd10mg	1 x tablet 10mg	€ 1.63 per tablet	Medicijnkosten.nl (3)
Clopidogrel alternative 3 (PM, TIA -50%)	dipyridamol: 2dd200mg	4 x tablet 200mg	€ 0.25 per tablet	Medicijnkosten.nl (3)
Clopidogrel alternative overall	Assumed 50% ACS indication (prasugrel and ticagrelor) and 50% TIA (dipyridamol)			
5-FU 100% (GAS 2)	400mg/m^2 2x per month for 6 months	1 x vial 50mg/mL 20mL	€ 6.81 per dose	Medicijnkosten.nl (3)
5-FU 50% (GAS 1.5, 1)	200mg/m^2 2x per month for 6 months	1 x vial 50mg/mL 10mL	€ 3.40 per dose	Medicijnkosten.nl (3)
5-FU alternative (GAS 0.5, 0)	Assumed same cost as 5-FU 100%			
Irinotecan 100% (EM)	350mg/m^2 every 3 weeks	1 x vial 20mg/mL 25mL and 1 x vial 20mg/mL 5 mL	€ 856.25 per dose	Medicijnkosten.nl (3)

Irinotecan 70% (*28/*28, PM)	245mg/m^2 every 3 weeks	1 x vial 20mg/ml 25mL	€ 712.74 per dose	Medicijnkosten.nl (3)
Mercaptopurine 100%	1dd 1.5mg/kg	2 x tablet 50mg	€ 2.68 per tablet	Medicijnkosten.nl (3)
Mercaptopurine 50% (IM)	1dd 0.75mg/kg	1 x tablet 50mg	€ 2.68 per tablet	Medicijnkosten.nl (3)
Mercaptopurine 10% (PM)	1dd 0.15mg/kg	15mg/mL 1mL vial	€ 16.35 per dose	Medicijnkosten.nl (3)
Tioguanine 100% (EM)	1dd 0.3mg/kg	1 x capsule 21mg	€ 2.98 per capsule	Medicijnkosten.nl (3)
Tioguanine 75% (IM)	1dd 0.225mg/kg	1 x capsule 16mg	€ 2.75 per capsule	Medicijnkosten.nl (3)
Tioguanine 6% (PM)	1dd 0.018mg/kg	1 x capsule 10mg	€ 2.49 per capsule	Medicijnkosten.nl (3)

References:

(1) Van der Wouden, C.H. Assessing the implementation of pharmacogenomic panel-testing in primary care in the Netherlands utilizing a theoretical framework.
(2) *Cao universitair medische centra 2018-2020.* <https://www.nfu.nl/img/pdf/19.2084_Uitgave_2019_-_Cao_umc_NL_2018-2020_v8.pdf>. Accessed February 2020.
(3) *Medicijnkosten.nl*. <https://www.medicijnkosten.nl/>. Accessed February 2020.
